# A 2-oxa-spiro[5.4]decane scaffold displays neurotrophic, neurogenic and *anti*-neuroinflammatory activities with high potential for development as a versatile CNS therapeutic

**DOI:** 10.1038/s41598-017-01297-z

**Published:** 2017-05-04

**Authors:** Pranav Chintamani Joshi, Ramesh Samineni, Dwaipayan Bhattacharya, Bommana Raghunath Reddy, Lenin Veeraval, Tapatee Das, Swati Maitra, Abhipradnya Bipin Wahul, Shailaja Karri, Srihari Pabbaraja, Goverdhan Mehta, Arvind Kumar, Sumana Chakravarty

**Affiliations:** 1 Chemical Biology, CSIR- Indian Institute of Chemical Technology, Tarnaka, Uppal Road, Hyderabad, 500007 India; 2 Natural Products Chemistry, CSIR- Indian Institute of Chemical Technology, Tarnaka, Uppal Road, Hyderabad, 500007 India; 3 School of Chemistry, University of Hyderabad, Hyderabad, 500046 India; 4 CSIR- Centre for Cellular and Molecular Biology, Habsiguda, Uppal Road, Hyderabad, 500007 India; 5 Academy of Scientific and Innovative Research, New Delhi, India

## Abstract

Following our recent discovery of a new scaffold exhibiting significant neurotrophic and neurogenic activities, a structurally tweaked analogue, embodying a 2-oxa-spiro [5.4]decane framework, has been conceptualised and found to be more potent and versatile. It exhibits enhanced neurotrophic and neurogenic action in *in vitro*, *ex vivo* and *in vivo* models and also shows robust neuroprotection in mouse acute cerebral stroke model. The observed attributes are traceable to the predominant activation of the TrkB-PI3K-AKT-CREB pathway. In addition, it also exhibits remarkable anti-neuroinflammatory activity by concurrently down-regulating pro-inflammatory cytokines IL-1α and IL-6, thereby providing a unique molecule with a trinity of neuroactivities, i.e. neurotrophic, neurogenic and anti-inflammatory. The new chemical entity disclosed here has the potential to be advanced as a versatile therapeutic molecule to treat stroke, depression, and possibly other neuropsychiatric disorders associated with attenuated neurotrophic/ neurogenic activity, together with heightened neuroinflammation.

## Introduction

One of the major global health care challenges today is to tackle debilitating neurological and psychiatric disorders that affect mood or emotion, and cognitive functions^1, 2^. The hallmarks of these diverse brain and behaviour disorders are neurodegeneration and/or altered neuroplasticity. Recent evidence highlight compromised neurotrophic and neurogenic characteristics on one hand and heightened neuroinflammation, on the other hand, associated with many central nervous system (CNS) disorders. The mood disorders that include depression, anxiety, post-traumatic stress disorder (PTSD), etc., are chronic debilitating CNS disorders, which in the long run also appear to cause cognitive decline^3, 4^. The cognitive disorders, grouped together as ‘dementia’, include diseases as diverse as Alzheimer’s disease, frontotemporal dementia, vascular dementia (induced by acute and chronic vascular perturbations or ischemic stroke), etc., and are progressively neurodegenerative in nature^5–7^. Another debilitating neurodegenerative condition is the cerebral ischemic stroke and the resultant vascular dementia cases are approaching an alarming level worldwide largely due to the lifestyle changes^8, 9^. Acute ischemic stroke is one of the leading causes of death and long-term disability and the most unfortunate thing is the limited therapeutic interventions to minimise and restore ischemia-induced neural damage^8, 9^. This is also largely the case with diverse cognitive conditions mentioned above and there is hardly any new CNS therapeutic introduced in recent decades to treat neurodegenerative conditions.

The neural circuitries function properly when there is substantial support of neurotrophins such as nerve growth factor (NGF), brain derived neurotrophic factor (BDNF), neurotrophin 3 (NTF3), glial derived neurotrophic factor (GDNF), etc refs [10–12]. However, the optimal level of production of neurotrophins and their action needs to be sustained, stimulated and augmented during the advanced phase of individual’s life in order to cope up with stress, neuroinflammation (which appears to augment during ageing), and to resist the onset of neurodegeneration. In this regard, a small molecule chemical entity that can cross the blood-brain barrier (BBB) and repair and/or regenerate neurons is an attractive premise. Thus, there is an urgent need to explore and identify new chemical entities, natural or otherwise, with diverse molecular architectures and to evaluate their neurotrophic, neurogenic and anti-neuroinflammatory potential towards the development of versatile CNS therapeutics^10, 13^. In this context, small molecule natural products (SMNP’s) have been found to promote neurite growth and synaptic plasticity by up-regulating the activity of neurotrophins^13, 14^. These observations, besides providing useful leads for the development of therapeutic agents to slow down neurodegeneration and to treat related disorders, emphasise the pressing need for synthetic chemist’s intervention to design new scaffolds by amplifying natural product diversity. With such an objective in mind, we recently designed two novel spirocyclic compounds #1 and #2 that exhibited potent neuroactivity at low concentrations^14, 15^. We discerned that #1 was more neurotrophic than neurogenic and provided neuroprotection in a mouse stroke model. On the other hand, #2 was more neurogenic than neurotrophic and failed to provide neuroprotection in the same model in acute single dose^13, 15^. This potent neurotrophic and/or neurogenic activity, as well as *in vivo* neuroprotective ability, shown by one of the novel spirocyclic compounds, led us to further explore the therapeutic space around this scaffold by employing a tactic of lowering the ring size to induce rigidity, modulating hydrophobicity and improving the binding directionality. In this vain, four spirocyclic compounds (#3–#6) based on 2-oxa-spiro [5.4]decane system were synthesised. Preliminary screening of these four (#3–#6) new series spirocyclic compounds in a range of neuroactivity essays indicated that among them #3 displayed the best potency in diverse models used by us (i.e. *in vitro* cell lines, *ex vivo* mouse primary neurosphere culture and *in vivo* zebrafish and mouse brain). Thus, #3 seems to have the potential to advance as a therapeutic molecule to treat stroke, depression and may be other neuropsychiatric disorders where neurotrophic and neurogenic activity is severely compromised and there is much neuroinflammation. Efforts were also directed to unravel the mechanisms of action of the new spirocyclic entity (#3) at the cellular and molecular level.

## Results

**Figure 1:**
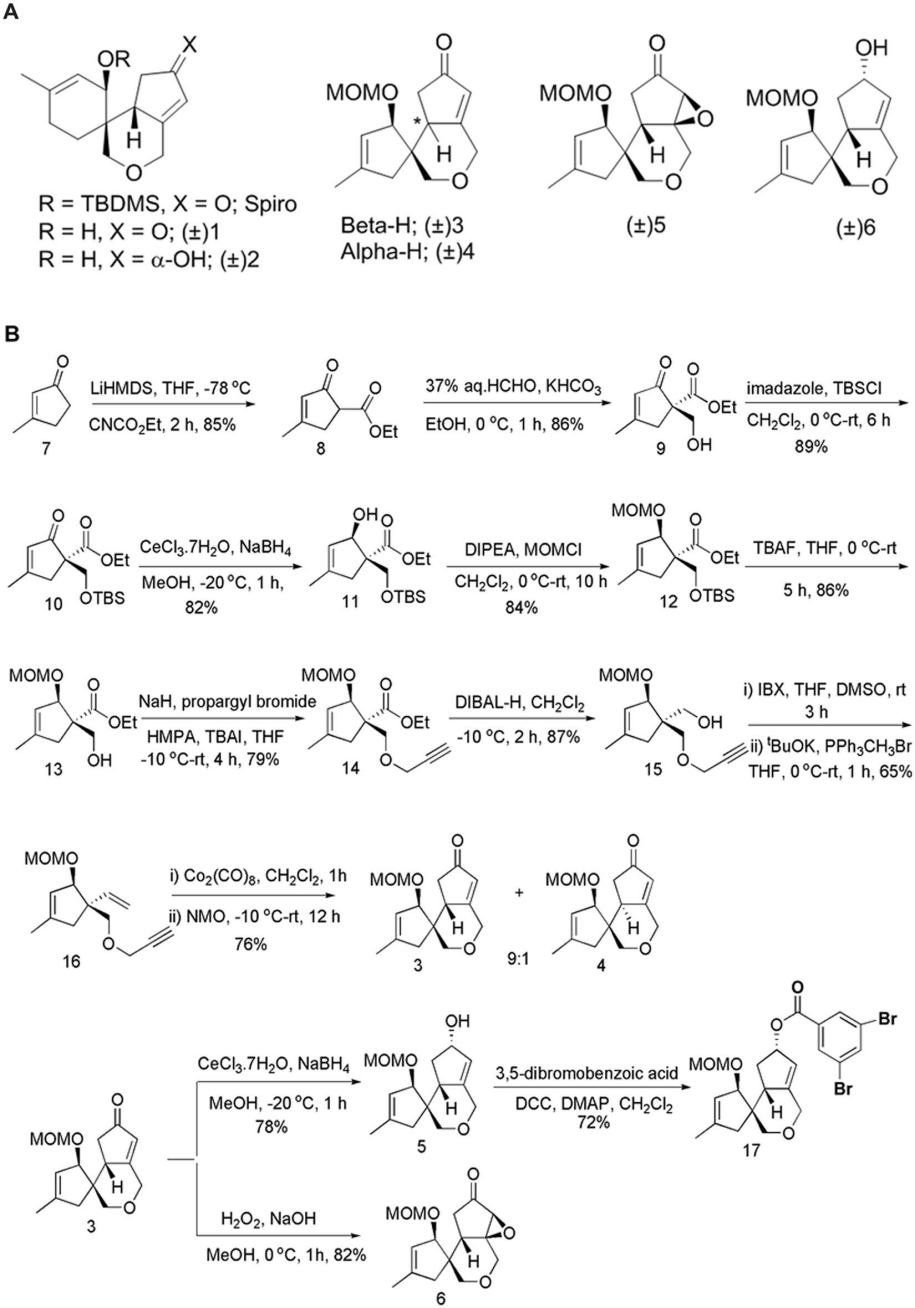
(**A**) Natural product inspired spiro[5.5]undecane and spiro[5.4]decane scaffolds.(**B**) Scheme followed for the synthesis of spirocyclic compounds 3, 4, 5 and 6.

The 2-oxa-spiro[5.4]decane based compounds #3–#6, as shown in Fig. 1A, were synthesised through a concise strategy shown in Fig. 1B and were thoroughly investigated for their neuroactivity and CNS therapeutic potential using *in vitro*, *ex vivo* and *in vivo* models.

### Evaluation of neuritogenic potential of compound #3 in inducing the transcription of several neurotrophic genes

**Figure 2:**
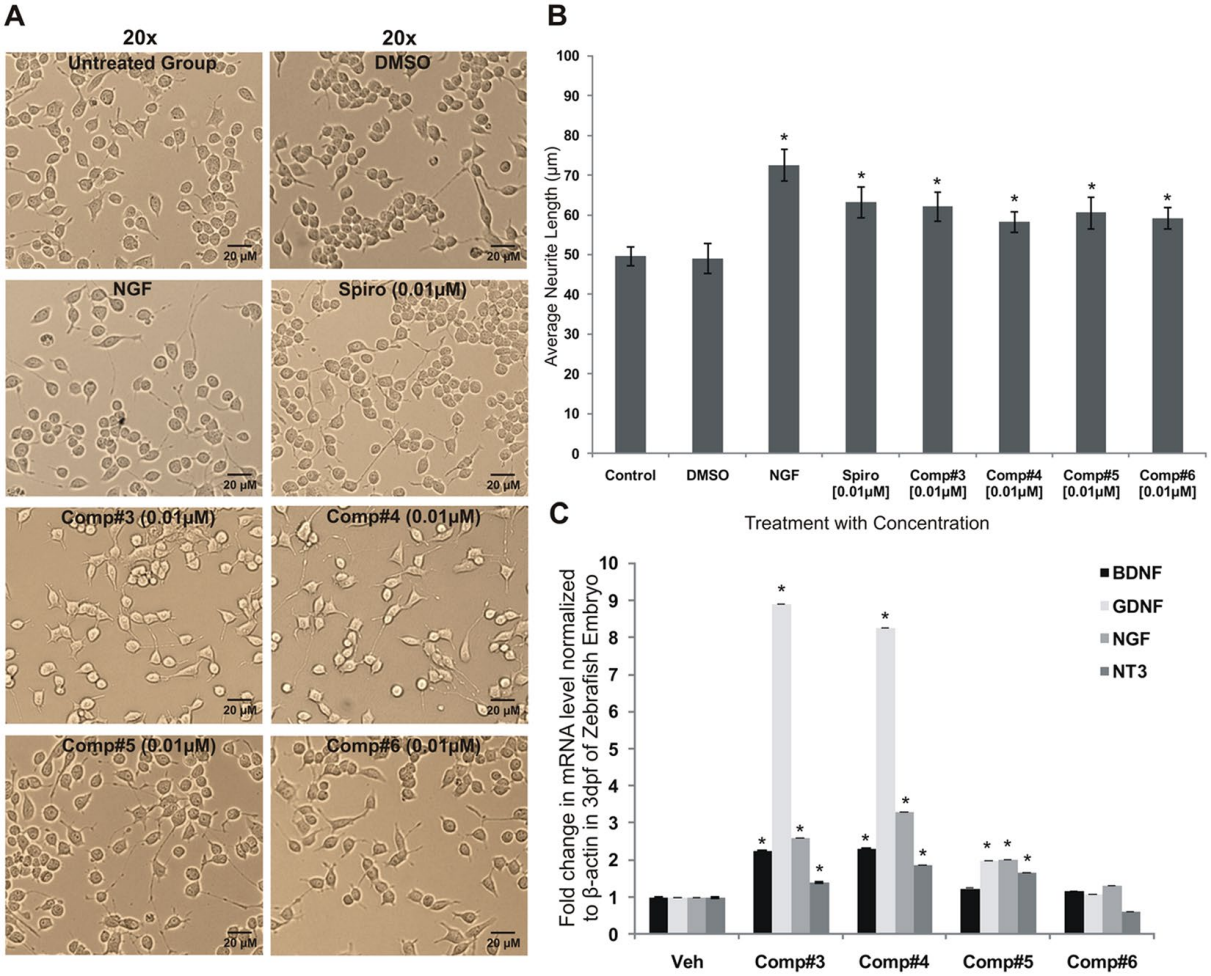
(**A**) Showing the morphometric analysis of neurite outgrowth of differentiated Neuro2A cells cultured for 24 h, followed by the treatment with NGF [200 ng/mL], DMSO [1%] and compounds #3–# 6 [0.01 μM]. (**B**) The tabulated and bar graph data are expressed as mean ± SEM where n = 60 and compared with the control. Note that all of them showed remarkable neurotrophic activity as compared to the untreated and DMSO-treated controls, *p < 0.05. (**C**) The relative level of expression in genes that code for neurotrophic factors like BDNF, GDNF, NGF and NT3 in 3dpf of Zebrafish larvae. Values are means + SEM, (n = 3), and each group containing 60 larvae, *p < 0.005.

The new chemical entities #3–#6 have initially tested for their neurotrophic action on mouse Neuro2A cells. The analysis of data showed that, all four compounds have significant neurotrophic activity at a dose as low as 0.01 μM, compared to the vehicle (DMSO) treatment control or untreated control (*p < 0.05) (Fig. 2A, 2B). After the potential neurotrophic activity was discovered in Neuro2A cells for these novel compounds, a reverse transcriptase-real time polymerase chain reaction (RT-qPCR) study was performed on the zebrafish embryo samples treated with the effective neurotrophic dose (1.0 μM for zebrafish embryo). It was found that only #3 and #4 could induce a significant level of transcription in most of the neurotrophic genes such as BDNF, GDNF, NGF and NT3, as evident in Fig. 2C.

From the dose-response study [10 μM to 0.01 μM] the optimum concentration was found to be as low as 0.01 μM for displaying significant neurotrophicity in Neuro2A cells, as shown in Suppl. Fig. 1B. Similarly, the cell viability was evaluated following the treatment of cells with these compounds, using MTT assay and the analysis of the data suggested no toxicity [shown in the Suppl. Fig. 1A].

### Evaluation of neurogenic potential of compound #3 in *ex vivo* and *in vivo* systems

**Figure 3:**
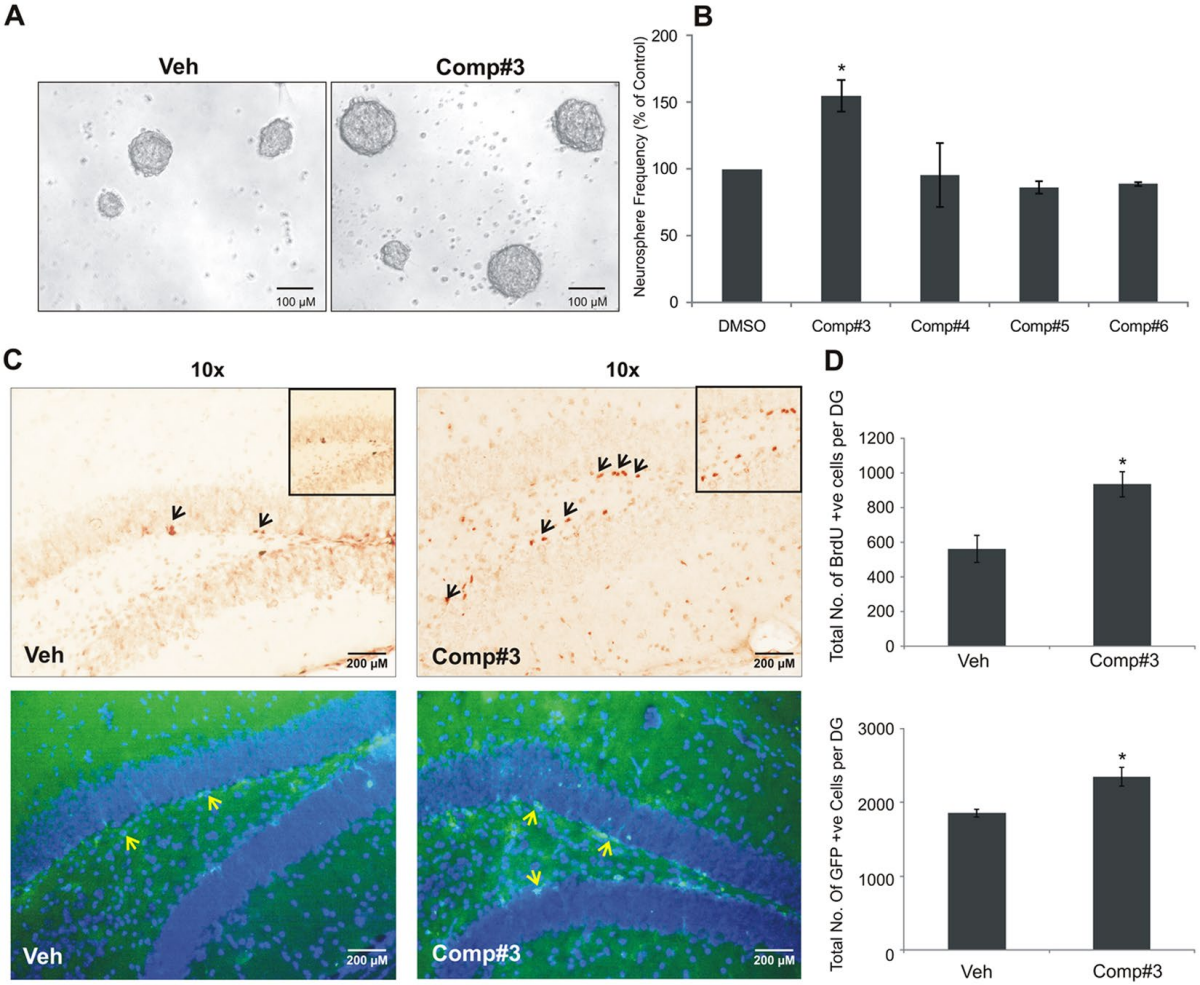
Neurosphere assay (**A,B**): Treatment of neural precursor cell population from postnatal (P2–3) mouse hippocampus by novel compounds in *ex vivo* assay shows the neurogenic potential only in #3; results are expressed as mean ± SEM, where neurosphere frequency (>100 μm size) is represented as percentage of control (n = 3/group); *p < 0.05 compared with the control (student’s t-test). (**C,D**) Shown are representative photomicrographs of Nestin +ve cells (C, top panel) and BrdU +ve cells (C, bottom panel) from vehicle -(DMSO 1%) and compound treated groups. (**D**) Number of Nestin +ve and BrdU +ve cells in the SGZ at the border of the granule cell layer (n = 4–5/group), *p < 0.05 compared with vehicle (student’s t-test).

After the identification of potent neurotrophic property, an attempt was made to explore the proneurogenic potential of the compounds. Initially, we performed *ex vivo* neurogenic assay using neural stem/progenitor cells (NSCs/NPCs) in primary culture, from the post natal day 2 (PND 2) mouse hippocampal dentate gyrus (DG) region. To obtain the optimum effective proneurogenic dose of the compound/s we counted the number of moderately good size of neurospheres formed by the highly proliferating NSCs/NPCs, at different concentrations of the compound/s (Suppl. Fig. 2). The data analysis suggested that #3 was highly proneurogenic in *ex vivo* mouse neurosphere assay (Fig. 3A,B) at 0.01 μM concentration and showed significantly high rate of proliferation of NSCs/NPCs or neurogenesis than #4–#6 of the series, as well as the earlier reported spiro compound #2^15^. Next, the proneurogenic potential of #3 was evaluated *in vivo* in nestin-GFP mice. Nestin-GFP mice were divided into two groups and injected with #3 (1 mg/kg; i.p.) and vehicle for 7 consecutive days, which was followed by daily injection of BrdU (50 mg/kg; i.p.) 30 min after the administration of the compound and vehicle. At the end of the treatment period, the animals in both the groups were intracardially perfused with 4% paraformaldehyde and the brain from each animal were taken out, post-fixed and sliced in cryomicrotome. The sliced brain sections were processed for the immunohistochemical localisation of Nestin-GFP and BrdU +ve cells in the neurogenic region of mouse hippocampus. Upon the quantitative analysis of Nestin-GFP +ve and BrdU +ve cells in the neurogenic DG of the mouse hippocampus, it was observed that #3 indeed had remarkably increased the turnover of NSCs/NPCs, as compared to the vehicle-treated mice (*p < 0.05). Note the higher level of neurogenesis in non-fluorescent based immunohistochemistry, as evident by significantly high BrdU +ve cells (upper panel in Fig. 3C,D) in #3-treated samples. Similarly, nestin population also showed a significant increase, as evident by the count of GFP +ve cells (lower panel in Fig. 3C,D).

However, BrdU method of quantifying the highly proliferative neural stem cell population in the telencephalon region of developing fish brain somehow did not yield conclusive result upon the treatment with compounds #3 and #4, except that the 3dpf Zebrafish embryos showed a trend of increased neurogenesis, compared to that treated by the vehicle (Suppl. Fig. 3).

### Evaluation of neuroprotective efficacy of compound #3 in mouse against global ischemia-induced cell death through neurotrophic and anti-neuroinflammatory action

**Figure 4:**
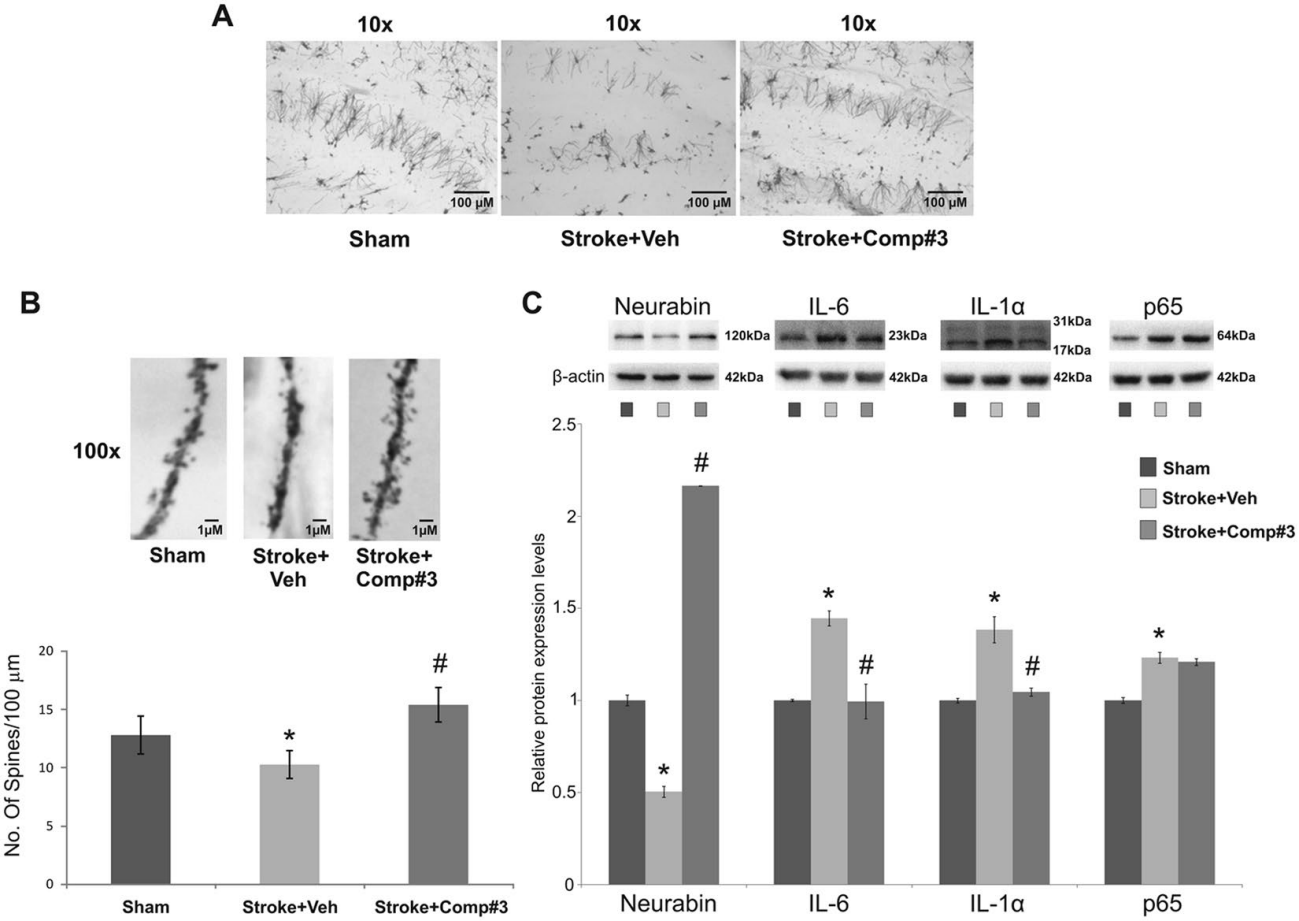
(**A,B**) Photomicrographs and the respective bar graph showing spine density in sham, vehicle and #3 treated group after BCCAo at 10x and 100x magnification. The compound #3 treated group showed protection again spine loss following BCCAo. (**C**) Showing immunoblot of neurabin, IL1α, IL6 and p65 (inflammatory markers) protein expression in the hippocampus. Lanes were loaded with 30 μg of protein. Expression was compared using densitometry. Densitometry results represent markers: β-actin ratios. Values are represented as mean ± SE with n = 3; p ≤ 0.05 indicates significant post hoc differences between sham vs vehicle group represented by (*) and vehicle vs #3 treated group, while represented by (^#^) by two-tailed student’s t-test. (n = 4–5/group).

For evaluating the neuroprotective action of the compound, experiments were performed in a mouse model of global ischemia with and without #3. It is pertinent to state here that the protein encoded by IL-1α gene is one of the widely known critical mediators of the neuroinflammatory response^16^. Our promising entity #3 showed noticeable neuroprotection against stroke-induced disruption in synaptic connectivity within the hippocampus, one of the affected brain regions (Fig. 4); the data is also supported by the significantly high level of neurabin 2 protein in hippocampus, a marker shown to be positively correlated with the enhanced synaptic connectivity in cortex^17^ (Fig. 4A–C).

Most of the neuropsychiatric disorders have been shown to be associated with neuroinflammation^18^ and so it was imperative to examine whether the neuroprotection by #3 was also due to its anti-inflammatory action. Consequently, the western blotting experiment was performed to see changes at the protein level of some crucial inflammatory markers in mouse hippocampi samples from both stroke and non-stroke groups. We found high levels of IL-1α and IL-6 in the affected ischemic region following an acute ischemic attack, which were significantly down in the group treated with #3. However, another inflammatory marker p65 of the nuclear factor kappa B (NFkB) pathway, failed to show any regulation by #3 (Fig. 4C). Further, we also checked spine density and neurabin 2 levels in other affected brain areas (striatum and prefrontal cortex). Interestingly, similar trend was observed in the prefrontal cortex, while treatment with #3 failed to exhibit any improvement in striatal spine density and neurabin 2 levels (Suppl. Fig. 4).

### Evaluation of antidepressant and anxiolytic efficacy of compound #3 using zebrafish model

**Figure 5:**
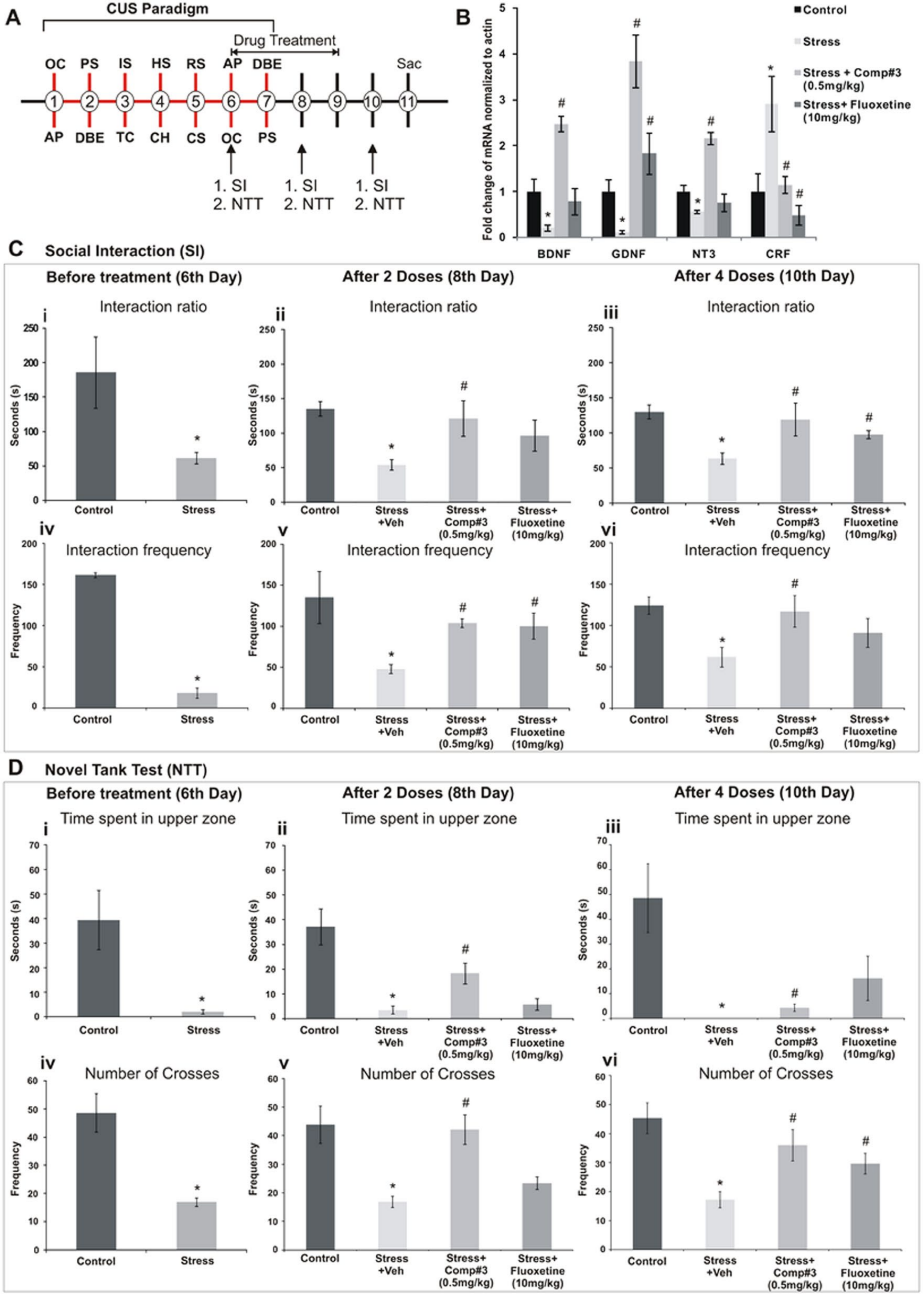
Antidepressant assay in adult zebrafish: CUS-induced behavioural compromise and altered neurotrophic profile. Schematic representation of the paradigm (**A**). Effect of the #3 and fluoxetine treatments on the social interaction test (**C**), Novel tank test (**D**) and mRNA levels of the neurotrophic factors (**B**). OC- Over Crowding, AP- Alarm Pheromone, PS- Predator Stress, DBE- Dorsal body exposure, IS- Isolation Stress, TC- Tank Change, HS- Heat Stress, CH- Chasing, RS- Restrain Stress, CS-Cold stress, SI- Social Interaction, NTT- Novel tank test. Values are represented as mean ± SE with n = 3; p ≤ 0.05 indicates significant post hoc differences between control vs stress group represented by (*) and stress vs treatment groups, while represented by (^#^) by two-tailed student’s t-test. (n = 18–20).

The neurogenic, neurotrophic and anti-neuroinflammatory efficacy of the compound (*vide supra*) motivated us to evaluate the antidepressant and/or antianxiety potential too, using a modified version of our previously reported chronic unpredictable stress (CUS) paradigm in Zebrafish^19^. After chronic stress paradigm as described in Fig. 5A, the social interaction (SI, for depression) and novel tank tests (NTT, for anxiety) were performed, with or without the treatment with #3 (0.5 mg/kg; i.p.). The outcome of the social interaction test, i.e. a decrease in interaction ratio and interaction frequency, a hallmark of depression that was observed in CUS group, was ameliorated in the CUS group treated with #3 (Fig. 5C). However, treatment with a widely used antidepressant fluoxetine failed to induce recovery from depression. This might be due to a brief (4 day) treatment period used in our CUS paradigm, as most of the antidepressants used in clinics including fluoxetine show their efficacy in animal models only when given for a long period of 2–3 weeks.

Similarly, in the novel tank test, the time spent in the upper zone by the CUS exposed fish got significantly reduced as compared to that by the control fish, indicating anxiety (Fig. 5D). The recovery from anxiety was quicker upon treatment with #3, than with the antidepressant and anxiolytic fluoxetine (Fig. 5D). Additionally, the expression of genes that code for neurotrophic factors such as BDNF, GDNF, NT3 were found to be diminished in the telencephalonic region of the fish brain in CUS group as compared to that in control group, showed significant recovery with the compound treatment after CUS. However, a brief period of fluoxetine treatment failed to restore the levels of the neurotrophins in telencephalon (Fig. 5B). Interestingly, the level of the transcript of stress responsive gene CRF in this brain region got significantly attenuated after the treatment with #3 and fluoxetine (10 mg/kg; i.p.), from the higher level found in the CUS group (Fig. 5B).

### Insights into the molecular mechanisms underlying the neurotrophic and neurogenic actions of compound #3

**Figure 6:**
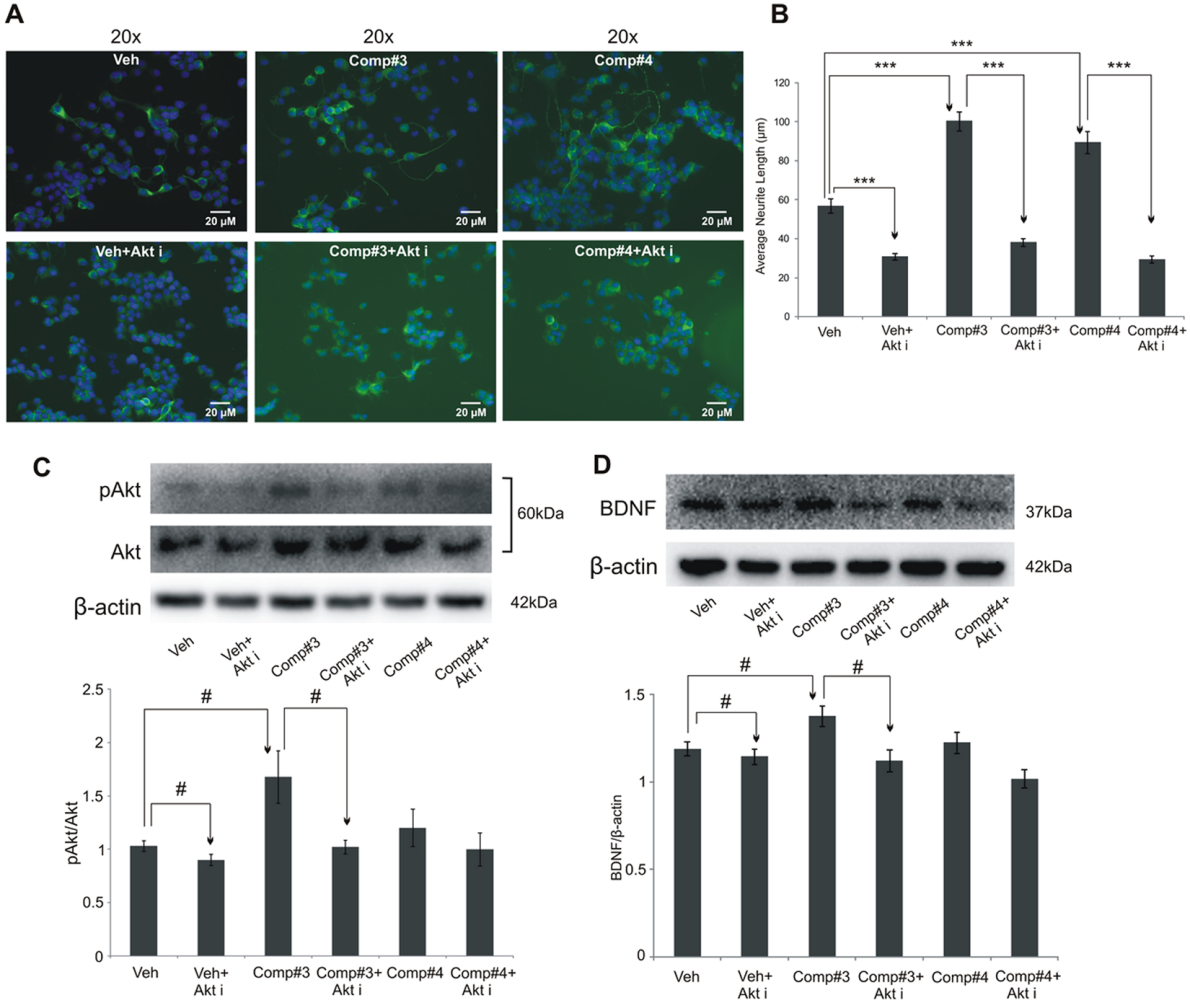
Mechanistic studies to uncover the mechanism of compounds’ neurotrophic action *in vitro* using AKT inhibitor (Akt i). Morphometric analysis of neurite outgrowth in differentiated Neuro2Acells treated with #3(0.01 μM) and #4(0.01 μM), pretreated (with or without AKT i, LY294002) (20 μM). Results were analysed by one-way ANOVA (with posthoc Tukey test) (**A,B**). Protein expression of pAKT-AKT (**C**) and BDNF (**D**) in Neuro2A cells treated with #3(0.01 μM) and #4(0.01 μM). Values are represented as mean ± SE with n = 3; Results analysed by either one-way ANOVA followed by post hoc Tukey test (**A,B** and **E,F**) represented as (*) or two-tailed unpaired student’s t-test for pairwise comparison between various groups represented as (^#^) where ^#^p < 0.05 and ***p < 0.001.

**Figure 7:**
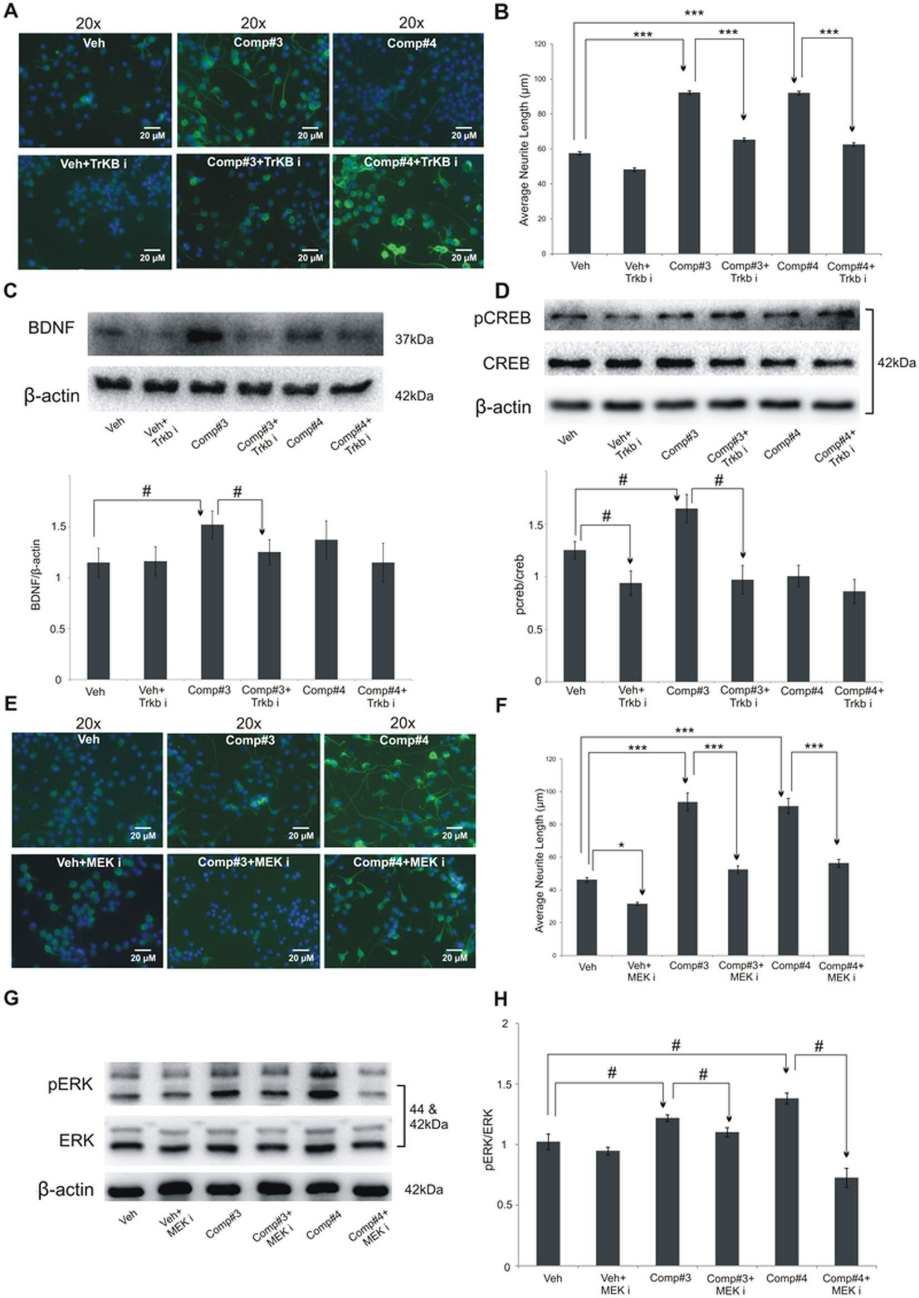
Mechanistic studies of compounds *in vitro* with MEK and TrkB inhibitor. Morphometric analysis of neurite outgrowth in differentiated Neuro 2A cells treated with #3(0.01 μM) and #4(0.01 μM), pre-treated with or without MEK and TrkB inhibitor, PD98059 (20 μM) and ANA-12 (10 μM), respectively (**A,B** and **E,F**). Protein expression of BDNF (**C**) and pCREB-CREB (**D**) in Neuro2A cells treated with #3(0.01 μM) and #4(0.01 μM) in TrkB inhibitor pre-treated cells. Similarly, protein expression of pERK-ERK (**G,H**) was evaluated in MEK inhibitor pre-treated Neuro2A cells. Values are represented as mean ± SE with n = 3; Results analysed by either one-way ANOVA followed by post hoc Tukey test (**A,B** and **E,F**) represented as (*) or two-tailed unpaired student’s t-test for pairwise comparison between various groups represented as (^#^) where ^#^p < 0.05 and ***p < 0.001.

To probe the molecular mechanisms involved in the neurotrophic action of #3, a number of studies were conducted. The results of initial *in vitro* assays showed that compound #3 acted through the TrkB-MEK-ERK pathway, like what was shown by us for compounds #1 and #2^15^. There are reports that TrkB-PI3K-AKT pathway also plays a role in neurptrophic activity^15, 20, 21^. So, using the inhibitor for this pathway it was observed that, the neurotrophic function of #3 is mediated via PI3K-AKT pathway too (Figs 6A,B and 7E,F). It is pertinent to mention here that, while performing experiments to probe the mechanism of #3, #4 was also included since both showed similar neuritogenic properties at *in vitro* level. We report here that, the neuritogenesis induced by both #3 and #4 was significantly attenuated when Neuro2A cells were pre-treated with specific inhibitors of these signalling pathways, PI3K-AKT (by LY294002) and MEK-ERK (by PD98059). Further studies at protein level to uncover the mechanistic details indicated the regulation of #3 induced neurotrophic activity via BDNF up-regulation in pAKT-AKT pathway, with no such indicative activity in case of #4 (Fig. 6C,D). Furthermore, unlike #3, the neurotrophic activity of compound #4 was blocked when the inhibitor of MEK-ERK pathway was used, and not as much when the inhibitor of PI3K-AKT was used (Fig. 7E,F). However, both #3 and #4 induced neurotrophic activity seemed to be regulated via MEK/ERK pathways as both the compounds exhibited significant changes in pERK/ERK protein expression levels (Fig. 7G,H).

Further, attenuation in the downstream marker pCREB/CREB as well as in BDNF upon pre-treatment with TrkB inhibitor, ANA-12 (Fig. 7C,D). Analysis of the results with/without TrkB inhibitor indicated^15^ a possible regulation of #3 induced neurotrophicity via TrkB receptor (Fig. 7A,B).

It is well known that both the signalling pathways (MEK-ERK and PI3K-AKT) play a key roles in transducing proliferative signals from membrane bound receptors^22^. Based on the possibility that crosstalk could be occurring between the PI3K and ERK1/2 pathways and to find out the predominant pathway involved in the action shown by #3, further investigation was done. The protein samples were generated after treating the cells with the inhibitors of MEK as well as PI3K-AKT pathways individually, along with a third group pre-treated with inhibitors for both the pathways simultaneously. The analysis of results from this experiment showed that the levels of pCREB/CREB, a common downstream molecule in both the signaling pathways, changed both in the combination (LY294002+ PD98059) group as well as PI3K-AKT (LY294002) inhibitor-treated group to the comparable extent, while no significant change was observed in the MEK inhibitor (PD98059) -treated group. This led us to reasonably conclude that the PI3K-AKT is the prime regulatory pathway for the neurotrophic action of #3 (Suppl. Fig. 5).

## Discussion

Most of the neuropsychiatric disorders including neurodegenerative ones are characterised by attenuated neurotrophic and/or neurogenic condition, in addition to elevated neuroinflammation^10, 23–26^. So, the search for a effective pharmacophore in an effort to develop CNS therapeutics to treat debilitating brain and behaviour disorders as diverse as dementia, stroke, depression and related disorders, that affect individual’s cognitive ability to an extent that they become dependable and takes huge toll in socio-economic term, has been there for quite some time^15, 27, 28^. Encouraged by the promise of two natural product paecilomycine A inspired compounds #1 and #2 based on a novel 2-oxa-spiro[5.5] undecane scaffold^15^ (where #1 proved to be more neurotrophic than neurogenic while #2 turned out more neurogenic than neurotrophic and #1 showed efficacious neuroprotection in acute ischemic stroke model in mouse), we amplified this project to gauge the therapeutic potential of four novel compounds (#3–#6) based on a framework where the scaffold had been tweaked through ring contraction of one of the rings. Our *in vitro* investigations on Neuro2A cells and *ex-vivo* proneurogenic assays using mouse hippocampal neural progenitor cell cultures showed that #3 possesses good neurogenic potential, in addition to robust neurotrophic capability. While evaluating the proneurogenic action of these new series compounds, #3 was found to induce significant upregulation in the proliferation of neural progenitor cells, as evident by moderate to big sized neurospheres in *ex vivo* neurosphere assay. Further, investigation using a transgenic nestin-GFP mouse model and chronic BrdU administration helped to uncover significantly high proneurogenic action of #3, as evident by 126% increase in nestin-GFP +ve cells and 166% increase in BrdU +ve cells in the hippocampal dentate gyrus, following the systemic treatment with #3 (1 mg/kg, i.p. for 7 days) in comparison to the vehicle-treated group. Furthermore, acute administration of #3 resulted in exceptional level of neuroprotection in an acute global ischemia mouse model (BCCAo), as evidenced by a decrease in pro-inflammatory IL-1α and IL-6 and an increase in enhanced synaptic connectivity marker neurabin 2 in the compound-treated ischemic group, in comparison to the level in the vehicle-treated ischemic group.

Depression, anxiety and related mood disorders are also associated with attenuated BDNF level^29, 30^ and neurogenesis^31^, together with neuroinflammation^18, 32, 33^. We tried to assess the antidepressant and anxiolytic efficacy of #3 using a slightly modified version of CUS model in Zebrafish we established in the lab^19^. Treatment with #3 (0.5 mg/kg, i.p. injection for four days, Refer Fig. 7A) could successfully restore the mood disturbance in zebrafish, i.e. helped in recovery from depression and anxiety-like phenotype in fish. Thus, our novel compound #3 is not only efficacious in a neurological condition like the ischemic stroke but also in a psychiatric condition like depression and related affective disorders.

Once we uncovered the neurotrophic, neurogenic and neuroprotective potential of compound #3, we started to elucidate the molecular mechanism underlying its potent action. So, first of all, it was assessed whether #3 treatment could result in the induction of transcription of neurotrophic factors BDNF, GDNF, NGF and NT3 in Zebrafish larvae. Both compounds #3 and #4 could induce a significantly high level of transcription of the genes that code for these neurotrophic factors (Fig. 2C). Finally, to get an insight into the signalling pathways that are involved in the neurotrophic action of the novel compound #3, we investigated TrkB-MEK-ERK-CREB and TrkB-PI3K-AKT-CREB pathways, the signalling pathways that have been reported to mediate the neurotrophic function downstream the activation of the neurotrophin receptor TrkB^18–20^. The *in vitro* cell culture experiments using the inhibitors of these selected pathways showed that #3 and #4 activate one or both these signalling pathways for the neurotrophic activity. It was observed that #3 could induce downregulation of pAKT and pERK to significant level following the treatment of cells with the specific inhibitors of the PI3K-AKT and MEK pathways, respectively. However, the predominant pathway through which #3 shows its neurotrophic action is PI3-AKT. Unlike #3, the neurotrophic activity of compound #4 appears to be through MEK-ERK pathway only, like we previously reported for compounds #1 and #2^15^.

In summary, the present study reports that the newly crafted molecule #3 displays potent neuroactivity at low concentration, without any apparent toxicity. It exhibits potent neurotrophic, neurogenic as well as anti-neuroinflammatory action *in vivo* in mouse brain and provides neuroprotection in a stroke model on acute single dosing. The compound also showed potent anxiolytic and antidepressant action in the zebrafish depression and anxiety model. Thus, our findings infer that the newly crafted compound #3 has a significantly amplified neuro-activity profile. It has the potential to be developed as a versatile therapeutic molecule for stroke, depression, and possibly other neuropsychiatric disorders characterised with compromised neurotrophic and/or neurogenic activity and concurrent neuroinflammation.

## Materials and Methods

### Synthesis of 2-oxa-spiro [5.4]decane compounds (#3–#6)

The new series of molecules #3 - #6 (as shown in Fig. 1A) were synthesised from commercially available 3-methyl-2-cyclopenten-1-one (7) as delineated in Fig. 1B.

Mander’s reagent mediated α-carboethoxylation of 7 under kinetic control yielded 8 and further hydroxymethylation with aq.formaldehyde provided alcohol 9. TBDMS protection as 10 followed by Luche’s reduction furnished the allylic alcohol 11. The newly generated hydroxyl group was protected as the corresponding MOM ether 12 and exposed to *n*-tetrabutylammonium fluoride (TBAF) to obtain desilylated alcohol 13. Propargylation of the hydroxyl group in 13 was achieved with NaH-HMPA to yield 14. To set up the intramolecular Pauson-Khand reaction, the ester functionality in 14 was transformed to an olefin 16 in a three step sequence involving DIBAL-H reduction to 15, IBX oxidation and Wittig homologation with methyltriphenylphosphonium bromide to furnish 16. Pauson-Khand reaction in ene-yne 16 in the presence of NMO furnished diastereomeric mixture (9:1) of tricyclic spiro-fused enones 3 and 4. Luche’s reduction and base mediated epoxidation in the major enone 3 led to the allylic alcohol 5 and the epoxide 6, respectively. The structural configuration of the compounds synthesized was determined with the help of X-ray analysis after derivatization of 5 to the corresponding solid 3,5-dibromobezoic acid derivative 17. Our further attempts to deprotect MOM moiety ended up in decomposition of the substrate molecule 6 and therefore it was deployed as such. The four 2-oxa-spiro [5.4]decane based compounds 3–6, were evaluated for their CNS activities using various systems/models.

### Cell Culture

Mouse Neuroblastoma cells (Neuro2A cells) were obtained from the American Type Culture Collection (ATCC) and were maintained as reported earlier^15^. Compounds were diluted in DMSO to different concentrations for the treatment where final DMSO concentration was not more than 1%, safe for the cell growth and viability.

### Animals

For *ex vivo* neurosphere assay, 2–3 days old C57BL/6 mouse pups were used. For *in vivo* assays three day post-fertilization (3 dpf) Zebrafish embryos, adult, male mice either C57BL/6 or Nestin-GFP transgenic mice, were used as required in various experiments. The wild type strain of Zebrafish was maintained as reported earlier^15, 34^. Mice were maintained in the institutional animal house facility, maintained at 25 °C ± 2 °C with a 12 h: 12 h light-dark cycle. Food and water to animals were available *ad libitum*. All animal procedures were carried out in “accordance” with approved guidelines of the Institutional Animal Ethics Committees [mouse protocol no. IAEC/CCMB/29/2013-14, and the Zebrafish protocol no. IICT/CB/SC/281114/30]. These protocols strictly follow the guidelines of CPCSEA, Government of India.

### Neurosphere assay

Mouse hippocampal neural progenitor cell (NSC) cultures were carried out according to previous reports^15, 35^. In brief, the pallet of single cell suspension was resuspended in Neurocult basal medium with proliferation supplements (Stem cell technologies) and plated at a density of 1000 cells/well in 96-well plate. Compounds #3–#6 were added individually to designated wells after 24 h of plating. The number of moderately growing neurospheres (>100 um) was counted after 5–6 days in culture. The experiment was repeated thrice where neurospheres from 5 wells were counted for each dose of compounds and the data were plotted in the graph.

### Neurogenesis in Zebrafish and mouse

The published protocols to assess the neurogenic efficacy of compounds were followed for both the species^15, 36^. Briefly, for BrdU labelling in the developing fish brain, 4 days post fertilisation (dpf.) zebrafish larvae were incubated in 10 mM BrdU (Sigma) in E3 media, fixed and processed for immunofluorescence (IF) labelling using mouse anti-BrdU antibody (1:500) with Alexa 488-conjugated secondary antibody (1:1000). The images were captured using stereomicroscope (Leica).

Similarly to assess the hippocampal neuronal proliferation, the Nestin-GFP transgenic mice were given an injection of #3 (1 mg/kg, i.p.) once daily for a period of 7 days, with a control group administered the saline injection in a similar manner. Both the groups were also injected once with BrdU (50 mg/Kg, i.p.), as described earlier and processed further^15^. The 30 µm thick hippocampal sections labelled with anti-mouse GFP antibody (Abcam, 1:500) and goat anti-rabbit alexa fluor 488 secondary antibody (Thermo fisher, 1:500), mounted with nuclear stain DAPI. The GFP +ve cells were quantified by stereological cell counting method as previously reported^15, 37^. For BrdU immunostaining, the sections were developed with diaminobenzidine (DAB) before mounting in DPX to count BrdU +ve cells in the hippocampus.

### Ischemic stroke and the treatment with compounds

Acute global ischemia was induced surgically by Bilateral Common Carotid Artery occlusion (BCCAo) in C57BL/6 male adult mice as per published protocols^15, 38^. Fifteen minutes before inducing ischemia #3 was injected (10 mg/Kg, i.p.). Reperfusion was done after 5–7 min of occlusion and after 24 h of BCCAo, the animals were sacrificed and the brain was taken out for Golgi-Cox histochemical staining.

### Golgi-Cox Staining

As reported earlier^15, 39, 40^, brain coronal sections of 50 μm were cut using Vibratome (OTS-4500 Harvard Apparatus, USA) and further processed for spine study. For analyses, 25 dendritic segments (50 µm each) were randomly selected from each group to measure the spine density using ImageJ software.

### Immunocytochemistry

For immunocytochemistry (ICC), cells after 24 h in culture with various treatments were processed for the immunostaining as previously reported^15^. Briefly, the PFA fixed cells were permeabilised and blocked followed by overnight incubation with anti β-III tubulin (Abcam, 1:1000) and further with goat anti-mouse IgG conjugated to alexa Flour 488 (Molecular Probes, 1:500). Images were captured using a Motic AE31 microscope.

### Western blotting

#### *In vivo* samples

The brain tissue samples were processed as described in previous report^41^. Briefly, 30 μg of total protein from each sample was electrophoresed on 10% SDS-PAGE gel and transferred to PVDF membrane. After blocking, the membrane was incubated overnight with appropriate primary antibodies: anti-p65 (Abcam, 1:1000), anti-β-actin (Abcam1:50000), anti-IL-1alpha (Abcam, 1:200), anti-IL6 (Abbiotec, 1:500), anti- neurabin 2 (Abcam, 1:1000) followed by incubation with the secondary HRP-conjugated antibody i.e. goat anti-mouse HRP (Santacruz, 1:10000) and goat anti-rabbit HRP (Santacruz, 1:5000) at room temperature for 2 h.

#### *In vitro* samples

Cells seeded at densities aforementioned in this section were and serum deprived for requisite interval and treated with respective pathway inhibitors (LY294002, PD98059, ANA-12) for an hour each following which #3 and #4 were administered. The protein was obtained from cell lysate, estimated with amido black method and electrophoresed. After blocking the PVDF membrane, incubated with primary antibodies BDNF (Abcam, 1:5000), pCREB (Millipore, 1:1000), CREB (Upstate, 1:500), anti-β-actin (Abcam, 1:5000), pAKT (CST, 1:1000), AKT (CST, 1:1000), pERK (Abcam, 1:1000) and ERK(Abcam, 1:1000) at 4 °C followed by incubation with HRP-conjugated anti-rabbit (1:5000) and anti-mouse (1:10000) at room temperature for 2 h.

Immunoreactivity was detected using the enhanced chemiluminescence kit (Thermo scientific) and visualised using Bio-Rad chemi Doc XRS+ imaging system with Image Lab acquisition and analysis software (CA, USA). Protein band densities were combined for each group normalised and assessed using Image-J software (NIH).

### RNA Isolation and Gene Expression

The total RNA was isolated from each set of treated groups (n = 60) containing larvae using TRIzol Reagent (Invitrogen) and cDNA was synthesised using Revert Aid H Minus First Strand cDNA Synthesis Kit, according to the manufacturer’s protocol. Primer sequences used for *in vivo* samples are available upon request. RT-qPCR was performed in triplicate by using SYBR Green PCR Master Mix Detection System (Applied Biosystems). The mRNA level was normalised by the level of the housekeeping gene β-actin. The relative level of gene expression was quantified for genes that code for BDNF, GDNF, NT3 and NGF using Δct method.

### Inhibitor Assays

In Neuro2A cells: 10,000 cells/cm^2^ were plated on with or without coverslips in a culture dish. After 24 h of incubation, cells were treated with PD98059 (MEK1/2 inhibitor) (20 μM), LY294002 (Phosphatidylinositol-3-kinase inhibitor) (20 μM) and ANA-12 (TrkB inhibitor) (10 μM). After 1 h of incubation with inhibitors, cells were treated with #3 and #4 (0.01 μM) and allowed for 24 h and 48 h of incubation. Cells were then fixed and used for neurite growth assay and ICC. For SDS-PAGE cell lysate was collected after 1 h of compound treatment.

### Antidepressant and anxiolytic assays using zebrafish model

The neurogenic and neurotrophic efficacy of the compound from the initial experimental results motivated us to evaluate the antidepressant/anti-anxiety potential using a previously reported chronic unpredictable stress (CUS) paradigm of our lab in zebrafish model^19^ with minor modifications. In brief, we let the fish undergo 5-days of chronic stress paradigm (as described in Fig. 5A), checked their behavioural status before and after the drug treatments by novel tank test (NTT) and social interaction test (SI) for the phenotypes of anxiety and depression respectively. For SI, a conspecific pink colour zebrafish served as a target in the interaction zone. Later, the fish were cold immobilised to dissect out telencephalons and processed immediately to assess mRNA levels of a stress responsive marker, CRF and well-reported depression fraternised neurotrophic factors such as BDNF, GDNF, NT3 and NGF by RT-qPCR. The results of the optimum treatment conditions [#3 treatment (0.5 mg/kg, i.p.); standard fluoxetine treatment (10 mg/kg, i.p.)] were independently compared with the CUS vehicle-treated group.

### Statistical Analysis

The results have been expressed as means ± S.E.M. The data were subjected to statistical analysis using one-way ANOVA followed by Tukey post hoc analysis and student’s t-test, according to the suitability.

## Electronic supplementary material

Supplementary Information

## References

[1] Collins PY (2011). Grand challenges in global mental health. Nature.

[2] Patel V, Flisher AJ, Hetrick S, McGorry P (2007). Mental health of young people: a global public-health challenge. The Lancet.

[3] Greenberg MS, Tanev K, Marin M-F, Pitman RK (2014). Stress, PTSD, and dementia. Alzheimer’s & Dementia.

[4] Qureshi SU (2010). Greater prevalence and incidence of dementia in older veterans with posttraumatic stress disorder. Journal of the American Geriatrics Society.

[5] Fuster-Matanzo A, Llorens-Martin M, Hernandez F, Avila J (2013). Role of neuroinflammation in adult neurogenesis and Alzheimer disease: therapeutic approaches. Mediators of inflammation.

[6] Daulatzai MA (2016). Fundamental role of pan-inflammation and oxidative-nitrosative pathways in neuropathogenesis of Alzheimer’s disease in focal cerebral ischemic rats. American journal of neurodegenerative disease.

[7] Freeman LC, Ting JP (2016). The pathogenic role of the inflammasome in neurodegenerative diseases. Journal of neurochemistry.

[8] Thurman, R. J., Jauch, E. C., Panagos, P. D., Reynolds, M. R. & Mocco, J. Four evolving strategies in the emergent treatment of acute ischemic stroke. *Emergency medicine practice* **14**, 1–26; quiz 26–27 (2012). 22872954

[9] Leng X (2016). Impact of collaterals on the efficacy and safety of endovascular treatment in acute ischaemic stroke: a systematic review and meta-analysis. Journal of neurology, neurosurgery, and psychiatry.

[10] Weissmiller AM, Wu C (2012). Current advances in using neurotrophic factors to treat neurodegenerative disorders. Translational neurodegeneration.

[11] Nagahara AH, Tuszynski MH (2011). Potential therapeutic uses of BDNF in neurological and psychiatric disorders. Nature reviews. Drug discovery.

[12] Cai J (2014). Potential therapeutic effects of neurotrophins for acute and chronic neurological diseases. BioMed research international.

[13] Jhelum P, Reddy RG, Kumar A, Chakravarty S (2016). Natural product based novel small molecules with promising neurotrophic, neurogenic and anti-neuroinflammatory actions can be developed as stroke therapeutics. Neural Regeneration Research.

[14] Mehta G, Samineni R, Srihari P, Reddy RG, Chakravarty S (2012). Diverted organic synthesis (DOS): accessing a new, natural product inspired, neurotrophically active scaffold through an intramolecular Pauson-Khand reaction. Organic & biomolecular chemistry.

[15] Chakravarty S (2015). A novel natural product inspired scaffold with robust neurotrophic, neurogenic and neuroprotective action. Scientific reports.

[16] Basu A, Krady JK, Levison SW (2004). Interleukin-1: a master regulator of neuroinflammation. Journal of neuroscience research.

[17] Nugent BM (2015). Brain feminization requires active repression of masculinization via DNA methylation. Nature neuroscience.

[18] Najjar S, Pearlman DM, Alper K, Najjar A, Devinsky O (2013). Neuroinflammation and psychiatric illness. Journal of neuroinflammation.

[19] Chakravarty S (2013). Chronic unpredictable stress (CUS)-induced anxiety and related mood disorders in a zebrafish model: altered brain proteome profile implicates mitochondrial dysfunction. PloS one.

[20] Tejeda GS, Díaz-Guerra M (2017). Integral Characterization of Defective BDNF/TrkB Signalling in Neurological and Psychiatric Disorders Leads the Way to New Therapies. International Journal of Molecular Sciences.

[21] Reichardt LF (2006). Neurotrophin-regulated signalling pathways. Philosophical Transactions of the Royal Society B: Biological Sciences.

[22] Dent P (2014). Crosstalk between ERK, AKT, and cell survival. Cancer biology & therapy.

[23] Hashimoto K, Shimizu E, Iyo M (2004). Critical role of brain-derived neurotrophic factor in mood disorders. Brain research reviews.

[24] Whitney NP, Eidem TM, Peng H, Huang Y, Zheng JC (2009). Inflammation mediates varying effects in neurogenesis: relevance to the pathogenesis of brain injury and neurodegenerative disorders. Journal of neurochemistry.

[25] Steiner B, Wolf SA, Kempermann G (2006). Adult neurogenesis and neurodegenerative disease. Regenerative medicine.

[26] Pieper AA (2010). Discovery of a proneurogenic, neuroprotective chemical. Cell.

[27] Mehta SL, Manhas N, Raghubir R (2007). Molecular targets in cerebral ischemia for developing novel therapeutics. Brain research reviews.

[28] Moskowitz MA, Lo EH, Iadecola C (2010). The science of stroke: mechanisms in search of treatments. Neuron.

[29] Nestler EJ (2002). Neurobiology of depression. Neuron.

[30] Duman RS, Monteggia LM (2006). A neurotrophic model for stress-related mood disorders. Biological psychiatry.

[31] Hill AS, Sahay A, Hen R (2015). Increasing Adult Hippocampal Neurogenesis is Sufficient to Reduce Anxiety and Depression-Like Behaviors. Neuropsychopharmacology: official publication of the American College of Neuropsychopharmacology.

[32] Wager-Smith K, Markou A (2011). Depression: a repair response to stress-induced neuronal microdamage that can grade into a chronic neuroinflammatory condition?. Neuroscience & Biobehavioral Reviews.

[33] Raison CL, Capuron L, Miller AH (2006). Cytokines sing the blues: inflammation and the pathogenesis of depression. Trends in immunology.

[34] Westerfield, M. The zebrafish book: a guide for the laboratory use of zebrafish Danio (Brachydanio) rerio. (Institute of Neuroscience, University of Oregon, [Eugene,OR, 1993).

[35] Yanpallewar SU (2010). Alpha2-adrenoceptor blockade accelerates the neurogenic, neurotrophic, and behavioral effects of chronic antidepressant treatment. The Journal of neuroscience: the official journal of the Society for Neuroscience.

[36] Yamaguchi M (2005). Analysis of neurogenesis using transgenic mice expressing GFP with nestin gene regulatory regions. Chemical senses.

[37] Lagace DC (2010). Adult hippocampal neurogenesis is functionally important for stress-induced social avoidance. Proceedings of the National Academy of Sciences of the United States of America.

[38] Murakami K, Kondo T, Kawase M, Chan PH (1998). The development of a new mouse model of global ischemia: focus on the relationships between ischemia duration, anesthesia, cerebral vasculature, and neuronal injury following global ischemia in mice. Brain research.

[39] Pilati N, Barker M, Panteleimonitis S, Donga R, Hamann M (2008). A rapid method combining Golgi and Nissl staining to study neuronal morphology and cytoarchitecture. The journal of histochemistry and cytochemistry: official journal of the Histochemistry Society.

[40] Gibb R, Kolb B (1998). A method for vibratome sectioning of Golgi-Cox stained whole rat brain. Journal of neuroscience methods.

[41] Reddy BR (2016). Sirtuin 1 and 7 mediate resveratrol-induced recovery from hyper-anxiety in high-fructose-fed prediabetic rats. Journal of biosciences.

