# Supplementary material for: A 2-oxa-spiro[5.4]decane scaffold displays neurotrophic, neurogenic and anti-neuroinflammatory activities with high potential for development as a versatile CNS therapeutic
Source: Sci Rep. 2017 May 4;7:1492. doi: 10.1038/s41598-017-01297-z (PMC5431446; doi:10.1038/s41598-017-01297-z)
Supplement: Supplementary file 1 — Supplementary Information [file 41598_2017_1297_MOESM1_ESM.pdf]

A 2-oxa-spiro[5.4]decane scaffold displays neurotrophic, neurogenic and anti-neuroinflammatory activities with high potential for development as a versatile CNS therapeutic

Pranav Chintamani Joshi[1^], Ramesh Samineni[2^], Dwaipayan Bhattacharya[1], Bommana Raghunath Reddy[1,5], Lenin Veeraval[1], Tapatee Das[1,5], Swati Maitra[1], Abhipradnya Bipin Wahul[1,5], Shailaja Karri [1], Srihari Pabbaraja[2,5]*, Goverdhan Mehta[3]*, Arvind Kumar[4,5]* and Sumana Chakravarty[1,5]*[#]

[1]Chemical Biology and [2]Natural Products Chemistry, CSIR- Indian Institute of Chemical Technology, Tarnaka, Uppal Road, Hyderabad 500007, India

[3]School of Chemistry, University of Hyderabad, Hyderabad-500046, India.

[4]CSIR- Centre for Cellular and Molecular Biology, Habsiguda, Uppal Road, Hyderabad 500007, India.

[5]Academy of Scientific and Innovative Research, New Delhi, India.

^ contributed equally to this work

* Senior authors

[#] Corresponding Author:

Dr. Sumana Chakravarty

Chemical Biology, CSIR- Indian Institute of Chemical Technology (IICT),

Tarnaka, Hyderabad-500007, India.

Email: sumanachak@iict.res.in, sumana98@gmail.com,

Phone: +91 040 27191856

Generated Report regarding BBB permeability using standard in silico software: http://www.cbligand.org/BBB/index.php

Compound Name	Structure	BBB Score
Spiro	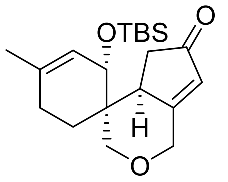	0.133
Comp#1	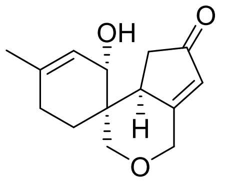	0.095
Comp#2	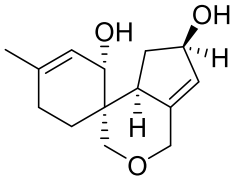	0.066
Comp#3	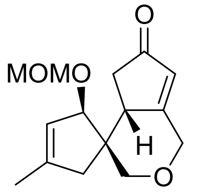	0.102
Comp#4	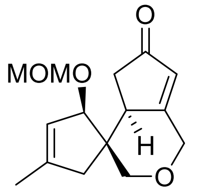	0.102
Comp#5	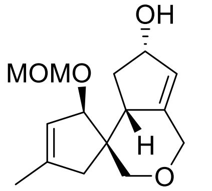	0.061
Comp#6	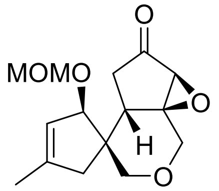	0.084

According to this method, all the above compounds are predictive of BBB permeability.

Suppl. fig.1

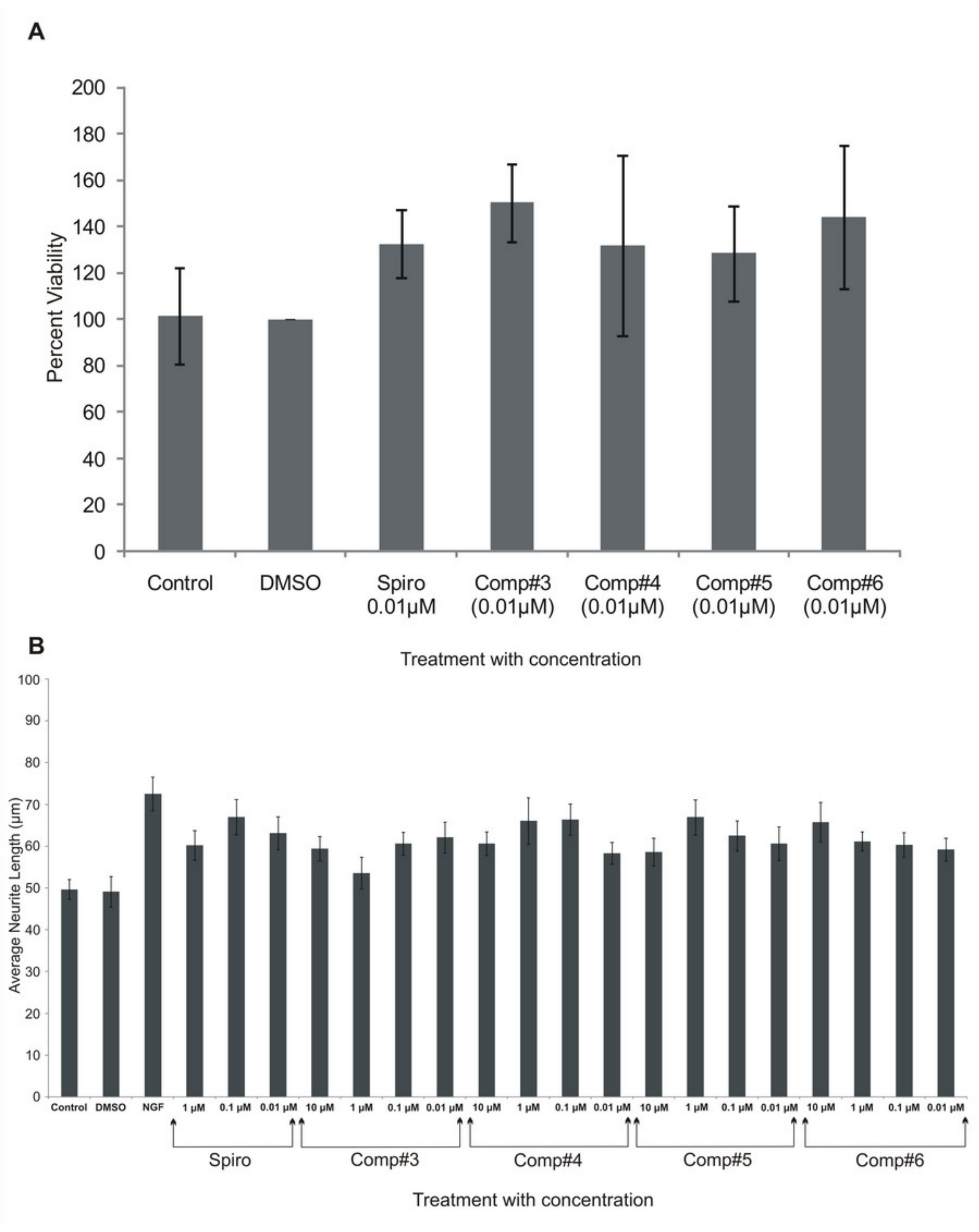

Suppl. fig.1: (A) Showing dose titration for *in vitro* experiments in Neuro2a cell line by MTT assay. (B) Showing the average neurite length for different concentrations of the compounds (spiro, #3- #6) from 0.01μM to 10.0 μ M along with NGF as a positive control.

Suppl. fig.2

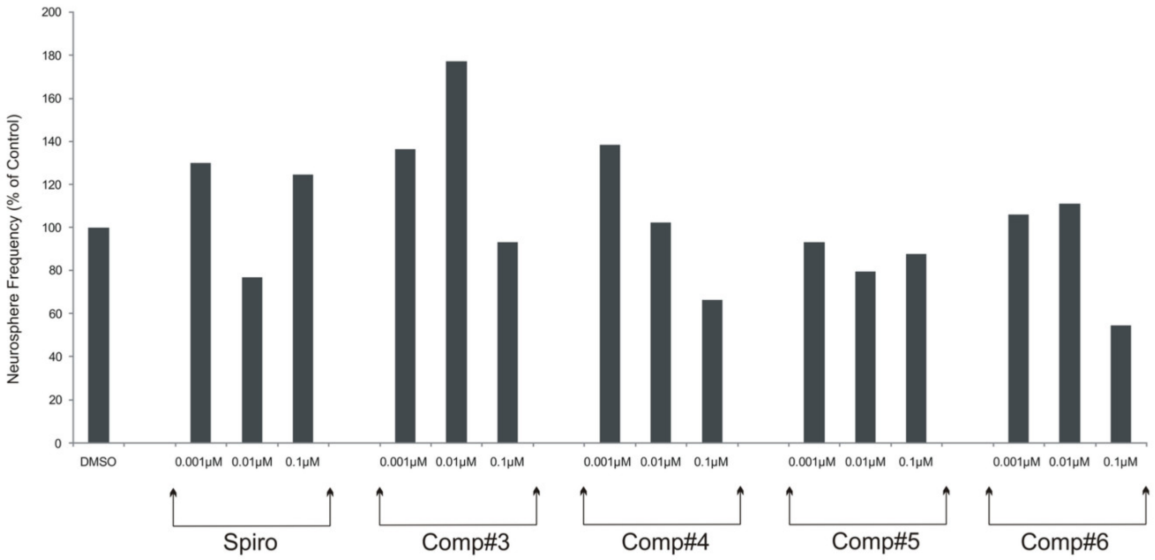

Suppl. fig.2: Dose titration for *ex vivo* experiment by using mouse hippocampal precursor cell populations subjected to different concentrations of the compounds (spiro, #3- #6) from 0.001µM to 1.0 µ M.

Suppl. fig.3

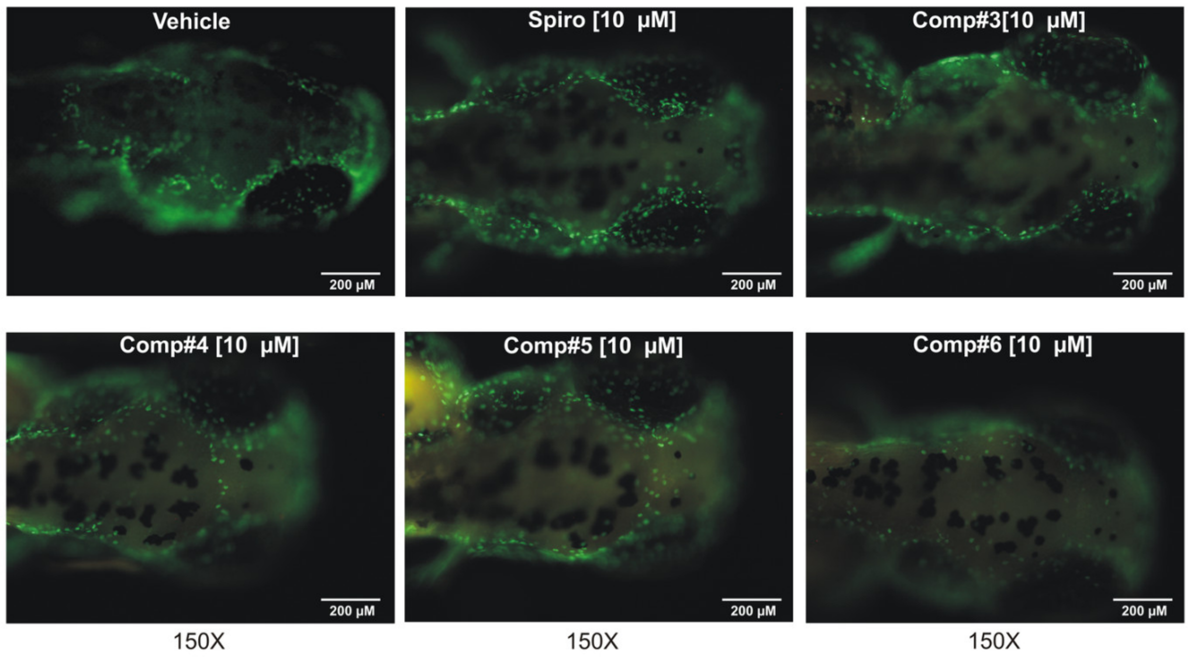

Suppl. fig.3: Whole mount immunofluorescence for BrdU in 4dpf zebrafish brain to assess the neurogenic activity of compounds (spiro, #3- #6).

Suppl. fig.4

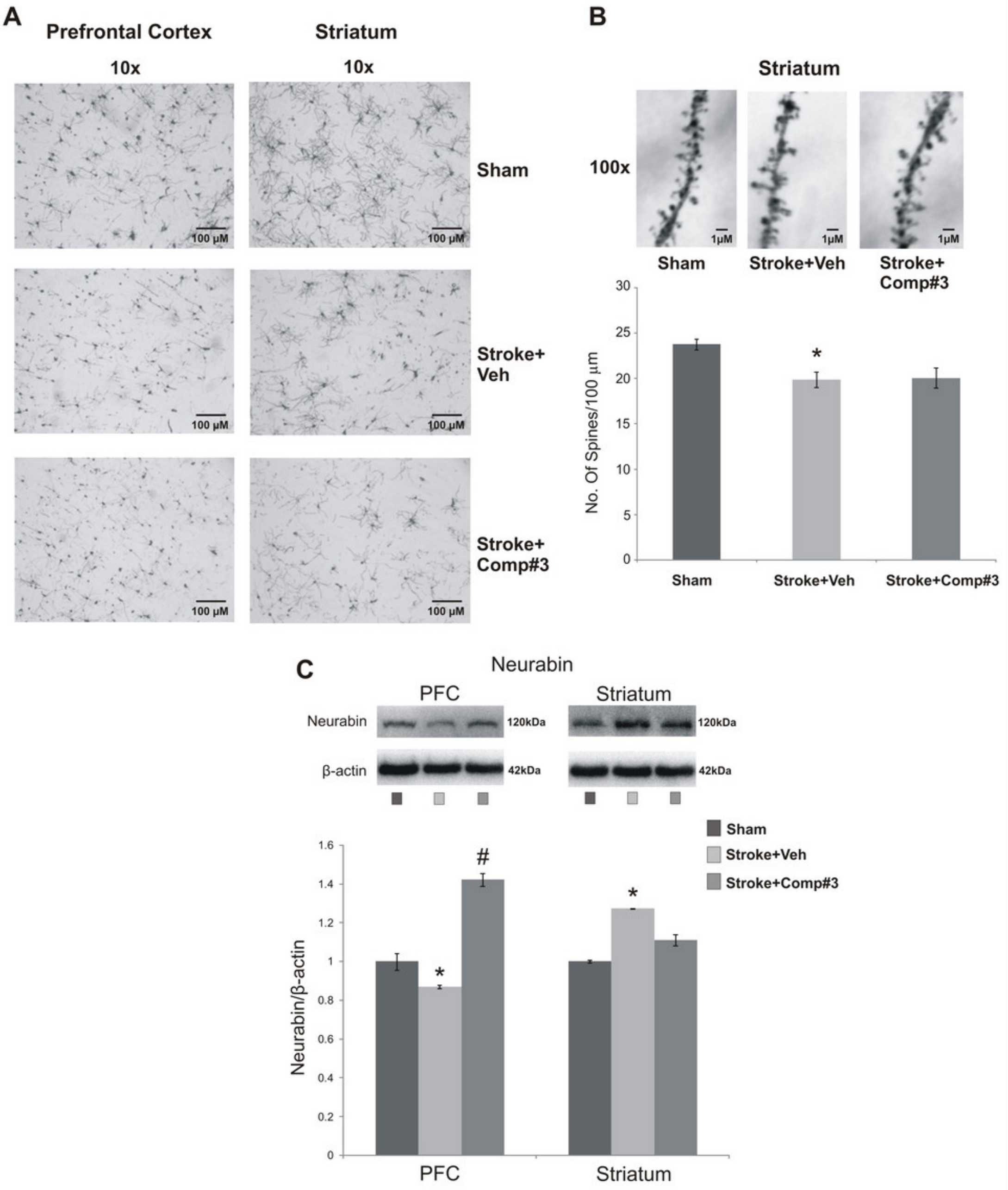

Suppl. fig.4: (A-B) Photomicrographs showing spine density in sham, vehicle and #3 treated group in PFC and striatum after BCCAo at 10x magnification (A). The #3 treated group could not show improvement in striatal spine density (B) whereas #3 indicated improvement in the spine density in PFC (not shown here). (C) Showing immunoblot and respective densitometric calculations of neurabin 2 in PFC and striatum. Densitometry results represent neurabin:β-actin ratios.Values are represented as mean ± SE with n=3; p≤ 0.05 indicates significant post hoc differences between sham vs vehicle group represented by (*) and vehicle vs #3 treated group, while represented by ($^{\#}$) by two-tailed student's t-test. (n= 4-5)

Suppl. fig.5

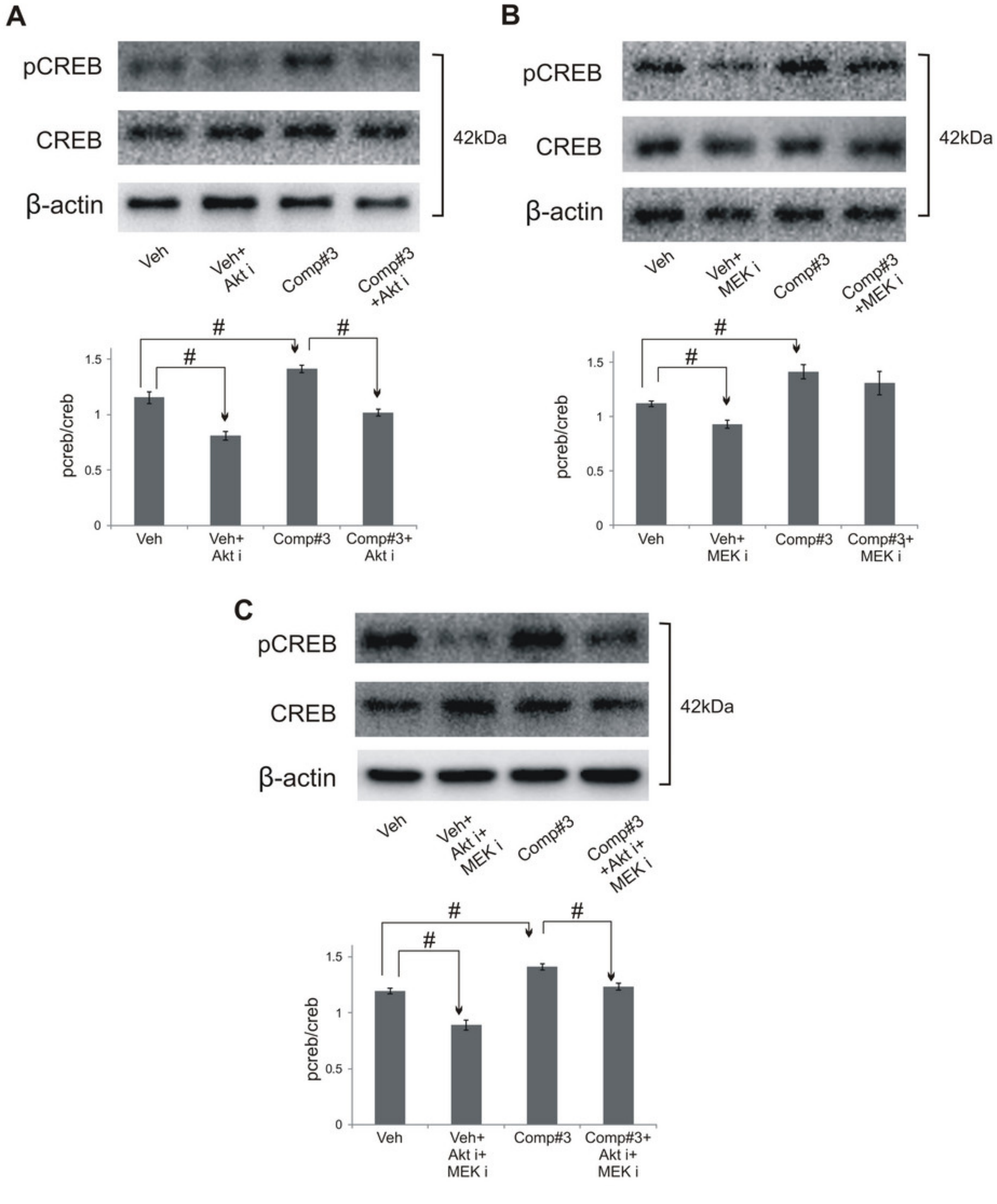

Suppl. fig.5: Mechanistic studies of compounds *in vitro* representing the potential of compound to act through Akt pathway. Representing protein expression of pCREB-CREB when the differentiated Neuro2A cells pretreated with or without AKT inhibitor, LY294002 (20 μM) (A), with or without MEK inhibitor, PD98059 (20 μM) (B) and combination of both Akt and MEK inhibitors (C). Values are represented as mean ± SE with n=3; where #p<0.05 indicates the significance analysed by two-tailed unpaired student's t-test for pairwise comparison between various groups.

Supporting information for the synthesis of compounds #3-#6:

General Methods:

FTIR spectra were recorded on KBr pellets $CHCl_3$/neat (as mentioned) and reported in wave number (cm^{-1}). ^1H NMR and ^{13}C NMR spectra were recorded in $CDCl_3$or $CDCl_3$ and CCl_4 as solvent on 300 MHz or 400 MHz 500 MHz spectrometer at ambient temperature. The coupling constant J is given in Hz and the chemical shifts are reported in ppm on scale downfield from TMS or $CDCl_3$ as internal standard and signal patterns are indicated as follows: s = singlet, d = doublet, dd = doublet of doublet, dt = doublet of triplet, t = triplet, q = quartet, qd = quartet of doublet, m = multiplet, br = broad. High (HRMS) resolution, *m/z* ratios are reported as values in atomic mass units. Mass analysis was done in ESI mode. All reagents were reagent grade and used without further purification unless specified otherwise. Solvents for reactions were distilled prior to use: THF, toluene and diethyl ether were distilled from Na and benzophenoneketyl; MeOH from Mg and I_2; CH_2Cl_2 from CaH_2. All air- or moisture-sensitive reactions were conducted under a nitrogen or argon atmosphere in flame-dried or oven-dried glassware with magnetic stirring. Reactions were monitored by thin-layer chromatography carried out on silica plates (silica gel 60 F254, Merck) using UV-light, iodine and anisaldehyde for visualization. Column chromatography was carried out using silica gel (60-120 mesh or 100-200 mesh) packed in glass columns. Technical grade ethyl acetate and petroleum ether used for column chromatography were distilled prior to use. All the compounds reported here are in racemic form.

Experimental Section:

Ethyl 4-methyl-2-oxocyclopent-3-enecarboxylate (8): To a stirred solution of 3-methyl cyclopentenone **7** (5.0 g, 52.08 mmol) in anhydrous tetrahydrofuran (THF) (50 mL) at -78 ^oC was added lithium bis(trimethylsilyl)amide(LiHMDS)(1 molar in tetrahydrofuran, 62.5 mL, 62.5 mmol) drop wise. The reaction was stirred for 30 min at the same temperature to this ethyl cyanoformate ($CNCO_2Et$) (5.3 mL, 57.3 mmol) was added drop wise and continued stirring until the starting material was disappeared in thin layer chromatography (2h), the reaction mixture was quenched by adding saturated ammonium chloride (40 mL) and water (20 mL), layers were separated and the aqueous layer was extracted with ethyl acetate (50 mL x 3), the combined organic extract was washed with brine (50 mL) and dried over sodium sulphate.Volatiles were removed to give crude oil, which was purified by silica gel column chromatography to afford ester **8** as pale yellow oil (7.4 g, 85%). R_f= 0.5 (7:3 hexane /ethyl

acetate); IR (neat): υ_{max} 1718, 1624, 1431, 1322, 1257, 1044, 839, 758 cm^{-1}; ^{1}H NMR (500 MHz, $CDCl_3$): δ 5.92-5.90 (m, 1H), 4.21 (q, *J* = 7.2 Hz, 2H), 3.45 (dd, *J* = 7.2, 2.9 Hz, 1H), 3.01-2.95 (m, 1H), 2.82-2.76 (m, 1H), 2.19 (s, 3H), 1.30 (t, *J* = 7.2 Hz, 3H); ^{13}C NMR (125 MHz, $CDCl_3$): δ 202.0, 178.7, 168.9, 128.5, 61.3, 52.5, 36.8, 19.1, 14.0; Mass (ESI): *m/z* $[M+Na]^+$ 191; HRMS (ESIMS) calcd for $C_9H_{12}O_3Na$ $[M+Na]^+$: *m/z* 191.0679; found: 191.0690.

Ethyl 1-(hydroxymethyl)-4-methyl-2-oxocyclopent-3-enecarboxylate (9): To a stirred solution of β-ketoester **8** (7.0 g, 41.66 mmol) in ethanol were added aqueous. formaldehyde (10 mL, 37-41% *w/v*) and potassium bicarbonate (10.4 g, 104.15 mmol) and allowed to stirr for 1 h at 27 °C. After completion of the reaction, the reaction mixture was diluted with water (50 mL) and extracted with ethyl acetate (100 mL x 3), the combined organic extract was washed with brine (50 mL) and dried over sodium sulphate. Volatiles were removed and the resulted crude compound was purified by silica gel column chromatography to afford alcohol **9** as yellow oil (7.1 g, 86%). R_f = 0.2 (7:3 hexane/ethyl acetate); IR (neat): υ_{max} 1713, 1623, 1430, 1377, 1268, 1045, 854, 762 cm^{-1}; ^{1}H NMR (500 MHz, $CDCl_3$): δ 5.93-5.92 (m, 1H), 4.20-4.16 (qd, *J* = 7.2, 1.1 Hz, 2H), 4.01-3.97 (m, 1H), 3.81-3.77 (m, 1H), 3.00 (d, *J* = 18.6 Hz, 1H), 2.90-2.82 (m, 2H), 2.22-2.20 (m, 3H), 1.24 (t, *J* = 7.2 Hz, 3H); ^{13}C NMR (125 MHz, $CDCl_3$): δ 203.9, 179.3, 170.4, 128.4, 64.5, 61.6, 61.5, 41.4, 19.3, 13.9; Mass (ESI): *m/z* $[M+Na]^+$ 221; HRMS (ESIMS) calcd for $C_{10}H_{15}O_4$ $[M+H]^+$: *m/z* 199.0965; found: 199.0975.

Ethyl-1-(((tert-butyldimethylsilyl)oxy)methyl)-4-methyl-2-oxocyclopent-3-ene-1-carboxylate (10): To a stirred solution of primary alcohol **9** (7.0 g, 35.35 mmol) in anhydrous dichloromethane (100 mL) at 0 °C were added imidazole (3.6 g, 53.02 mmol) and *tert*-butyldimethylsilyl chloride (6.4 g, 42.42 mmol) and stirred for 3 h. After completion of the reaction as indicated by thin layer chromatography, the reaction mixture was quenched by adding saturatedaqueous ammonium chloride (50 mL) and diluted with water (50 mL), layers were separated and the aqueous layer was extracted with dichloromethane (100 mL x 3), the combined organic extract was washed with brine (100 mL) and dried over sodium sulphate. Volatiles were removed to give crude oil, which was purified by silica gel column chromatography to afford **10** as pale yellow oil (9.8 g, 89%).R_f = 0.5 (9:1 hexane/ethyl acetate); IR (neat): υ_{max} 2954, 2932,1724, 1628, 1467, 1379, 1256, 1101, 843, 778 cm^{-1}; ^{1}H NMR (500 MHz, $CDCl_3$): δ 5.89-5.88 (m, 1H), 4.14 (q, *J* = 7.2 Hz, 2H), 4.00 (d, *J* = 9.6 Hz,

1H), 3.93 (d, *J* = 9.6 Hz, 1H), 3.06-3.01 (m, 1H), 2.81-2.76 (m, 1H), 2.18-2.16 (m, 3H), 1.20 (t, *J* = 7.2 Hz, 3H), 0.80 (s, 9H), 0.01 (s, 3H), -0.01 (3H); ^{13}C NMR (75 MHz, $CDCl_3$): δ 203.8, 179.0, 169.4, 128.6, 64.5, 61.9, 61.4, 41.1, 25.6 (3C), 19.3, 18.0, 14.0, -5.7 (2C); Mass (ESI): *m/z* $[M+Na]^+$ 335; HRMS (ESIMS) calcd for $C_{16}H_{28}O_4SiNa$ $[M+Na]^+$: *m/z* 335.1649; found: 335.1675.

Ethyl-1-(((tert-butyldimethylsilyl)oxy)methyl)-2-hydroxy-4-methylcyclopent-3-ene-1-carboxylate (11): The enone **10** (9.5 g, 30.45 mmol) was dissolved in methanol (100 mL) and cooled to -20 °C, to this cerium(III) chloride heptahydrate ($CeCl_3.7H_2O$) (10.9 g, 33.45 mmol) was added and stirred for 5 min after which sodium borohydride (1.27 g, 10.11 mmol) was added and stirred for 15 min. After completion of the reaction (by thin layer chromatography), the reaction mixture was quenched with saturated aqueous ammonium chloride (20 mL) and water (50 mL) and extracted with ethyl acetate (100 mL x 4). The combined organic extract was washed with brine (100 mL), and dried over sodium sulphate, evaporated to afford crude compound which was purified by column chromatography to obtain pure allylic alcohol **11** as pale yellow oil (7.8 g, 82%). R_f = 0.4 (9:1 hexane/ethyl acetate); IR (neat): υ_{max} 3462, 2955, 2932, 1729,1252, 1093, 842, 777 cm^{-1};^{1}H NMR (300 MHz, $CDCl_3$): δ 5.40-5.36 (m, 1H), 5.18-5.14 (m, 1H), 4.20-4.11 (m, 2H), 4.00 (d, *J* = 9.6 Hz, 1H), 3.88 (d, *J* = 9.6 Hz, 1H), 2.91 (d, *J* = 6.0 Hz, 1H), 2.60 (d, *J* = 17.0 Hz, 1H), 2.33 (d, *J* = 17.0 Hz, 1H), 1.73-1.70 (m, 3H), 1.26 (t, *J* = 7.2 Hz, 3H), 0.86 (s, 9H), 0.05 (s, 6H); ^{13}C NMR (75MHz, $CDCl_3$): δ 175.2, 142.0, 126.5, 81.2, 65.5, 60.8, 57.7, 43.3, 25.6 (3C), 18.0, 16.4, 14.2, -5.7, -5.8; Mass (ESI): *m/z* $[M+Na]^+$ 337; HRMS (ESIMS) calcd for $C_{16}H_{30}O_4SiNa$ $[M+Na]^+$: *m/z* 337.1806; found: 337.1823.

Ethyl-1-(((tert-butyldimethylsilyl)oxy)methyl)-2-(methoxymethoxy)-4-methylcyclopent-3-ene-1-carboxylate (12): To a stirred solution of allylic alcohol **11** (3.0 g, 9.55 mmol) in anhydrous dichloromethane (30 mL) at 0 °C were added *N*,*N*-diisopropylethylamine (2.5 mL, 14.3 mmol) and methoxymethyl chloride (1 mL, 12.4 mmol) and stirred for 10 h. After completion of the reaction as indicated by thin layer chromatography, the reaction mixture was quenched with sat ammonium chloride (10 mL) and water (20 mL), layers were separated and the aqueous layer was extracted with dichloromethane (50 mL x 3), the combined organic extract was washed with brine (40 mL) and dried over sodium sulphate. Volatiles were removed to give crude oil, which was purified by silica gel column

chromatography to afford **12** as pale yellow oil (2.94 g, 84%). R_f = 0.5 (9:1 hexane/ethyl acetate); IR (neat): υ_{max} 2932, 1728, 1445, 1198, 1096, 1034 cm^{-1}; ^1H NMR (300 MHz, $CDCl_3$): δ 5.34 (brs, 1H), 4.87 (brs, 1H), 4.79 (d, *J* = 6.6 Hz, 1H), 4.60 (d, *J* = 6.6 Hz, 1H), 4.14 (q, *J* = 7.2 Hz, 2H), 4.04 (d, *J* = 9.4 Hz, 1H), 3.70 (d, *J* = 9.3 Hz, 1H), 3.36 (s, 3H), 2.76 (d, *J* = 17.2 Hz, 1H), 2.39 (d, *J* = 17.2 Hz, 1H), 1.75 (s, 3H), 1.26 (t, *J* = 7.2 Hz, 3H), 0.85 (s, 9H), 0.02 (s, 3H), 0.01 (s, 3H); ^{13}C NMR (75MHz, $CDCl_3$): δ 175.2, 143.7, 124.3, 96.5, 85.5, 64.9, 60.7, 59.4, 55.2, 42.2, 25.7 (3C), 18.1, 16.7, 14.2, -5.6, -5.7; Mass (ESI): *m/z* $[M+Na]^+$ 381; HRMS (ESIMS) calcd for $C_{16}H_{30}O_4SiNa$ $[M+Na]^+$: *m/z* 381.2068; found: 381.2097.

Ethyl-1-(hydroxymethyl)-2-(methoxymethoxy)-4-methylcyclopent-3-ene-1-carboxylate (13): To a solution of compound **12** (2.9 g, 8.1 mmol) in anhydrous tetrahydrofuran (30 mL) was added tetrabutylammonium fluoride (9.72 mL, 9.72 mmol, 1 molar solution in tetrahydrofuran) and stirred for 5 h. After completion of the reaction as indicated by thin layer chromatography, the reaction mixture was quenched with saturated aqueousammonium chloride (10 mL), water (30 mL) and extracted with ethyl acetate (50 mL x 3), dried over sodium sulphate and evaporated. The compound was purified by silica gel column chromatography to give **13** as oil (1.7 g, 86%).R_f= 0.4 (4:1 hexane/ethyl acetate); IR (neat): υ_{max} 3450, 1710, 1425, 1352, 1262, 1140, 1040, 825, 777 cm^{-1}; ^1H NMR (300 MHz, $CDCl_3$): δ 5.36 (s, 1H), 5.14 (s, 1H), 4.75 (d, *J* = 6.4 Hz, 1H), 4.67 (d, *J* = 6.6 Hz, 1H), 4.17 (q, *J* = 7.2 Hz, 2H), 3.89-3.75 (m, 2H), 3.36 (s, 3H), 3.00 (t, *J* = 7.7 Hz, 1H), 2.55 (d, *J* = 17.0 Hz, 1H), 2.37 (d, *J* = 17.0 Hz, 1H), 1.71 (s, 3H), 1.25 (t, *J* = 7.2 Hz, 3H); ^{13}C NMR (75MHz, $CDCl_3$): δ 175.1, 142.6, 124.2, 96.6, 87.1, 65.3, 61.0, 57.9, 55.5, 42.8, 16.4, 14.1; Mass (ESI): *m/z* $[M+Na]^+$ 267; HRMS (ESIMS) calcd for $C_{12}H_{20}O_5Na$ $[M+Na]^+$: *m/z* 267.1203; found: 267.1227.

Ethyl-2-(methoxymethoxy)-4-methyl-1-((prop-2-yn-1-yloxy)methyl)cyclopent-3-ene-1-carboxylate (14): To a stirred solution of hydroxy ester **13** (1.6 g, 6.55 mmol) in tetrahydrofuran (20 mL) at -10 °C was added hexamethylphosphoramide (3 mL) and sodium hydride (525 mg, 13.11 mmol, 60% in mineral oil) and stirred for 10 min, to this propargyl bromide (1.01 mL, 9.83 mmol, 80% in toluene) and tetrabutylammonium iodide (20 mg) were added and stirred for 3 h. After completion of the reaction monitored by thin layer chromatography, the reaction mixture was quenched with saturated aqueousammonium chloride (5 mL), water (30 mL) and extracted with ethyl acetate (30 mL x 3). The combined

organic extract was washed with brine (30 mL) and dried over sodium sulphate, volatiles were removed to obtain crude compound which was purified by silica gel column chromatography to give **14** as yellow oil (1.46 g, 79%). R_f = 0.7 (4:1 hexane:ethylacetate); IR (neat): υ_{max} 3277, 2941, 2119, 1725, 1446, 1227, 1098 cm^{-1}; ^{1}H NMR (300 MHz, $CDCl_3$): δ 5.34 (m, 1H), 4.87 (s, 1H), 4.75 (d, *J* = 6.7 Hz, 1H), 4.61 (d, *J* = 6.7 Hz, 1H), 4.17 (q, *J* = 7.2 Hz, 2H), 4.12 (d, *J* = 2.2 Hz, 2H), 3.93 (d, *J* = 8.7 Hz, 1H), 3.66 (d, *J* = 8.7 Hz, 1H), 3.36 (s, 3H), 2.79 (d, *J* = 17.0 Hz, 1H), 2.48-2.43 (m, 1H), 2.39 (t, *J* = 2.3 Hz, 1H), 1.76-1.75 (m, 3H), 1.25 (t, *J* = 7.2 Hz, 3H); ^{13}C NMR (125MHz, $CDCl_3$): δ 174.8, 143.7, 124.0, 96.4, 85.8, 79.6, 74.2, 71.5, 61.0, 58.5, 57.5, 55.3, 42.4, 16.6, 14.1; Mass (ESI): *m/z* $[M+Na]^+$ 305; HRMS (ESIMS) calcd for $C_{15}H_{22}O_5Na$ $[M+Na]^+$: *m/z* 305.1359; found: 305.1343.

2-(Methoxymethoxy)-4-methyl-1-((prop-2-yn-1-yloxy)methyl)cyclopent-3-en-1-yl)methanol (15): The ester **14** (1.4 mg, 4.96 mmol) was dissolved in dichloromethane (20 mL) and cooled to -10 °C, to this diisobutylaluminum hydride (7.8 mL, 10.92 mmol, 20% *w/v* in toluene) was added dropwise and stirred for 1 h. After completion of the reaction as indicated by thin layer chromatography, the reaction mixture was quenched with saturated aqueous sodium potassium tartrate (20 mL) and diluted with dichloromethane (20 mL) and stirred for 1 h at 27 °C, layers were separated and organic layer was dried over sodium sulphate and evaporated to give crude alcohol which was purified by silica gel column chromatography to give alcohol **15** as pale yellow oil (1.04 g, 87%).R_f = 0.30 (4:1 hexane:ethylacetate); IR (neat): υ_{max} 3425, 2914, 2117, 1096, 1037cm^{-1}; ^{1}H NMR (300 MHz, $CDCl_3$): δ 5.42 (s, 1H), 4.70 (d, *J* = 6.6 Hz, 1H), 4.62 (d, *J* = 6.6 Hz, 1H), 4.44 (brs, 1H), 4.17 (d, *J* = 2.2 Hz, 2H), 3.86 (d, *J* = 8.8 Hz, 1H), 3.63 (d, *J* = 9.1 Hz, 1H), 3.60 (d, *J* = 10.7 Hz, 1H), 3.50 (d, *J* = 10.7 Hz, 1H), 3.37 (s, 3H), 2.45 (t, *J* = 2.2 Hz, 1H), 2.25 (d, *J* = 17.0 Hz, 1H), 1.98 (d, *J* = 17.0 Hz, 1H), 1.76 (s, 3H); ^{13}C NMR (75MHz, $CDCl_3$): δ 144.1, 124.5, 96.3, 85.1, 79.5, 74.6, 72.8, 68.0, 58.6, 55.2, 50.5, 42.2, 16.8; Mass (ESI): *m/z* $[M+Na]^+$ 263; HRMS (ESIMS) calcd for $C_{13}H_{20}O_4Na$ $[M+Na]^+$: *m/z* 263.1254; found: 263.1252.

3-(Methoxymethoxy)-1-methyl-4-((prop-2-yn-1-yloxy)methyl)-4-vinylcyclopent-1-ene (16): The alcohol **15** (1.0 g, 4.16 mmol) was dissolved in tetrahydrofuran (10 mL) and added drop wise to a pre-dissolved solution of 2-Iodoxybenzoic acid (1.75 g, 6.25 mmol) in dimethyl sulphoxide(5 mL) and stirred for 3 h at 27 °C. After completion of the reaction solids were filtered with the aid of diethyl ether (50 mL). The etheral layer was washed with

saturated aqueous sodium bicarbonate (10 mL x 2), cold water (15 mL x 3), dried over sodium sulphate and evaporated to give crude aldehyde (1 g), this compound was directly used for next reaction without further purification.

The aldehyde (1.0 g, 4.2 mmol) was dissolved in anhydrous tetrahydrofuran (10.0 mL) and the solution was added to a stirred solution of methylidenetriphenylphosphorane prepared from potassium *tert*-butoxide (941 mg, 8.4 mmol) and methyltriphenylphosphoniumiodide (3.75 g, 10.5 mmol) in tetrahydrofuran (40 mL) at 0 ^{o}C under inert atmosphere. The reaction mixture was stirred for 15 min at room temperature. After completion of the reaction (by thin layer chromatography), the reaction mixture was quenched with saturated aqueous ammonium chloride (10 mL), water (20 mL) and extracted with diethyl ether (30 mL x 3). The combined organic extract was washed with brine (20 mL), dried over sodium sulphate. Volatiles were removed under reduced pressure. The resulting residue was purified by column chromatography on silica gel to give enyne **16** (639 mg, 65 % over two steps) as pale yellow oil. R_f =0.50 (95:5 hexane/ethyl acetate); IR (neat): υ_{max} 3308, 2930, 2123, 1741, 1693, 1516, 1464, 1253, 1112, 841, 777, 633 cm^{-1}; ^{1}H NMR (300 MHz, $CDCl_3$): δ 6.02 (dd, *J* = 17.7, 11.0 Hz, 1H), 5.41 (brs, 1H), 5.11-5.04 (m, 2H), 4.71 (d, *J* = 6.6 Hz, 1H), 4.63 (d, *J* = 6.6 Hz, 1H), 4.42 (brs, 1H), 4.16 (d, *J* = 2.3 Hz, 2H), 3.74 (d, *J* = 8.9 Hz, 1H), 3.58 (d, *J* = 8.9 Hz, 1H), 3.37 (s, 3H), 2.47 (d, *J* = 16.4 Hz, 1H), 2.40 (t, *J* = 2.4 Hz, 1H), 2.16 (d, *J* = 17.1 Hz, 1H), 1.77 (s, 3H); ^{13}C NMR (75MHz, $CDCl_3$): δ 144.1, 143.2, 124.2, 112.6, 96.4, 88.0, 80.0, 74.1, 72.6, 58.6, 55.6, 52.21, 44.1, 16.9. Mass (ESI): *m/z* $[M+Na]^+$ 259; HRMS (ESIMS) calcd for $C_{14}H_{20}O_3Na$ $[M+Na]^+$: *m/z* 259.1305; found: 259.1302.

2-(Methoxymethoxy)-4-methyl-4a',5'-dihydro-3'H-spiro[cyclopentane-1,4'-cyclopenta[c] pyran]-3-en-6'(1'H)-one (3) and 2-(methoxymethoxy)-4-methyl-4a',5'-dihydro-3'H-spiro[cyclopentane-1,4'-cyclopenta[c]pyran]-3-en-6'(1'H)-one (4): To a solution of the enyne **16** (600 mg, 2.54 mmol) in anhydrous dichloromethane (30 mL), which was degassed by bubbling with nitrogen for 15 min, was added cobalt octacarbonyl (1.04 g, 3.05 mmol). The mixture was stirred for 1 h at room temperature and cooled to -10 ^{o}C and NMO (2.08 g, 17.78 mmol) was added, the reaction was stirred at 27 ^{o}Cfor 12 h. After completion of the reaction (by thin layer chromatography), the reaction mixture was filtered through a pad of celite with the aid of dichloromethane (10 mL x 3) and the filtrate was concentrated under reduced pressure. The residue was purified by column chromatography on silica gel to afford the tricyclic enones**3** (460 mg, semi solid) and **4** (50 mg, semi solid) in 76% yield. R_f= 0.4

(3:2 hexane/ethyl acetate);**3:** IR (neat):υ_{max} 2914, 1712, 1632, 1445, 1148, 1095, 1036 cm^{-1}; ^1H NMR (300 MHz, $CDCl_3$): δ 5.97 (br s, 1H), 5.41 (br s, 1H), 4.66-4.60 (m, 3H), 4.35 (br s, 1H), 4.15 (d, *J* = 13.6 Hz, 1H), 3.91 (d, *J* = 11.9 Hz, 1H), 3.65 (d, *J* = 11.7 Hz, 1H), 3.37 (s, 3H), 3.10 (d, *J* = 6.2 Hz, 1H), 2.43-2.30 (m, 2H), 1.96 (dd, *J* = 19.1, 2.5 Hz, 1H), 1.76-1.70 (m, 1H), 1.72 (s, 3H); ^{13}C NMR (100 MHz, $CDCl_3$): δ 208.0, 174.7, 144.8, 128.1, 124.7, 96.4, 88.8, 72.6, 66.7, 55.4, 50.5, 47.2, 39.8, 36.6, 16.7; Mass (ESI): *m/z* $[M+Na]^+$ 287; HRMS (ESIMS) calcd for $C_{15}H_{20}O_4Na$ $[M+Na]^+$: *m/z* 287.1254; found: 287.1270; **4:** IR (neat):υ_{max} 2924, 1712, 1632, 1444, 1148, 1095, 1035, 918 cm^{-1}; ^1H NMR (300 MHz, $CDCl_3$): δ 6.01 (s, 1H), 5.46 (s, 1H), 4.66-4.53 (m, 3H), 4.23-4.15 (m, 2H), 3.92 (s, 1H), 3.40 (d, *J* = 11.5 Hz, 1H), 3.30 (s, 3H), 2.97 (d, *J* = 5.7 Hz, 1H), 2.34-2.14 (m, 3H), 1.89 (d, *J* = 17.0 Hz, 1H), 1.75 (s, 3H); ^{13}C NMR (75MHz, $CDCl_3$): δ 207.6, 173.5, 144.9, 128.3, 126.0, 96.6, 82.0, 71.0, 66.2, 55.1, 49.5, 49.2, 45.7, 36.0, 16.7; Mass (ESI): *m/z* $[M+Na]^+$ 287; HRMS (ESIMS) calcd for $C_{15}H_{20}O_4Na$ $[M+Na]^+$: *m/z* 287.1254; found: 287.1241.

2-(Methoxymethoxy)-4-methyl-1',4a',5',6'-tetrahydro-3'H-spiro[cyclopentane-1,4'-cyclopenta[c]pyran]-3-en-6'-ol (5): The enone **3** (60 mg, 0.23 mmol) was dissolved in methanol (3 mL) and cooled to -20 ^oC, to this cerium(III) chloride heptahydrate ($CeCl_3.7H_2O$) (82 mg, 0.25 mmol) was added and stirred for 5 min after which sodium borohydride (10 mg, 0.25 mmol) was added and stirred for 15 min. After completion of the reaction (by thin layer chromatography), the reaction mixture was quenched with saturated aqueous ammonium chloride (2 mL), water (3 mL) and extracted with ethyl acetate (5 mL x 3). The combined organic extract was washed with brine (10 mL), dried over sodium sulphate, evaporated to afford crude compound which was purified by column chromatography to obtain pure allylic alcohol **5** as semi solid (47 mg, 78%). R_f= 0.3 (3:2 hexane/ethyl acetate); IR (neat):υ_{max} 3501, 2955, 2862, 1742, 1438, 1220, 1182, 1063 cm^{-1}; ^1H NMR (300 MHz, $CDCl_3$): δ 5.51 (m, 1H), 5.40 (s, 1H), 4.85 (br s, 1H), 4.65-4.59 (m, 2H), 4.38 (d, *J* = 13.0 Hz, 1H), 4.28 (br s, 1H), 3.96 (br s, 1H), 3.89 (d, *J* = 11.7 Hz, 1H), 3.48 (d, *J* = 11.5 Hz, 1H), 3.36 (s, 3H), 2.73 (t, *J* = 7.0 Hz, 1H), 2.42-2.32 (m, 2H), 2.07 (d, *J* = 17.2 Hz, 1H), 1.89 (brs, 1H), 1.76 (s, 3H), 1.17-1.09 (m, 1H); ^{13}C NMR (75MHz, $CDCl_3$): δ 145.2, 142.9, 126.2, 124.4, 96.2, 88.7, 76.3, 72.6, 66.5, 55.3, 50.9, 49.2, 40.7, 34.8, 16.8; Mass (ESI): *m/z* $[M+Na]^+$ 289; HRMS (ESIMS) calcd for $C_{15}H_{22}O_4Na$ $[M+Na]^+$: *m/z* 281.1410 found: 281.1413.

2-(Methoxymethoxy)-4-methyl-1',4a',5',6'-tetrahydro-3'H-spiro[cyclopentane-1,4'-cyclopenta[c]pyran]-3-en-6'-yl 3,5-dibromobenzoate (17): To a stirred solution of 3, 5-dibromo benzoic acid (15.6 mg, 0.056 mmol), dicyclohexylcarbodiimide(11.6 mg, 0.056 mmol) in dichloromethane(3 mL) at 0 °C were added alcohol **5** (10 mg, 0.038 mmol), 4-(Dimethylamino)pyridine(1.5 mg, 0.012 mmol) and continued stirring at 27 °C for 10 h. The reaction mixture was quenched with saturated aqueousammonium chloride (1 mL), water (2 mL) and extracted with dichloromethane (5 mL x 3). The combined organic extract was washed with brine, dried over sodium sulphate, evaporated to give crude compound which was purified by silica gel column chromatography to afford 17 as white solid (14 mg, 72%) which was readily crystallized in dichloromethane, hexane to give crystals.R_f = 0.60 (3:2 hexane/ethyl acetate); IR (neat):υ_{max} 3078, 2926, 1727, 1557, 1420, 1253, 1129, 743 cm^{-1}; ^1H NMR (300 MHz, $CDCl_3$): δ 8.07 (d, *J* = 1.8 Hz, 2H), 7.84 (t, *J* = 1.8 Hz, 1H), 5.93-5.88 (m, 1H), 5.66-5.63 (m, 1H), 5.44-5.41 (m, 1H), 4.64 (q, *J* = 6.7 Hz, 2H), 4.43 (d, *J* = 12.8 Hz, 1H), 4.32 (brs, 1H), 3.98 (d, *J* = 12.8 Hz, 1H), 3.92 (d, *J* = 11.7 Hz, 1H), 3.54 (d, *J* = 11.7 Hz, 1H), 3.38 (s, 3H), 2.88-2.83 (m, 1H), 2.59-2.48 (m, 1H), 2.41 (d, *J* = 17.8 Hz, 1H), 2.13 (d, *J* = 17.8 Hz, 1H), 1.76 (s, 3H), 1.51-1.43 (m, 1H); ^{13}C NMR (100 MHz, $CDCl_3$): δ 163.6, 146.6, 145.2, 138.2, 133.6, 131.2, 124.7, 123.0, 121.8, 96.3, 88.7, 80.8, 72.7, 66.4, 55.4, 51.2, 49.8, 40.8, 31.3, 16.9; Mass (ESI): *m/z* $[M+Na]^+$ 548; HRMS (ESIMS) calcd for $C_{22}H_{24}O_5Br_2Na$ $[M+Na]^+$: *m/z* 548.9883 found: 548.9843.

ORTEP Diagram of 17: CCDC number: 1486813.

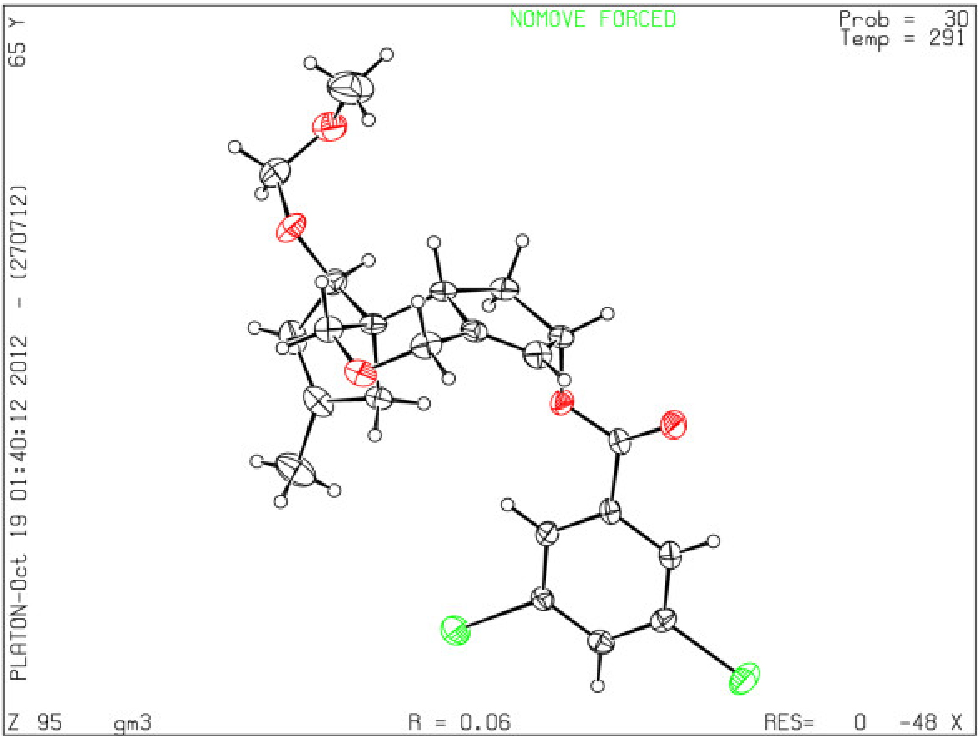

2-(Methoxymethoxy)-4-methyl-3',3a'-dihydro-5'H,7'H-spiro[cyclopentane-1,4'-oxireno[2',3':1,5]cyclopenta[1,2-c]pyran]-3-en-2'(1a'H)-one (6): To a stirred solution of enone**3**(50 mg) in methanol (3 mL) at 0 °C were added hydrogenperoxide (0.5 mL, 30% w/v in water) and 6N sodium hydroxide (1 mL). After stirring at 27 °C for 2 h the reaction mixture was diluted with ethyl acetate (5 mL) and washed with brine containing sodium thiosulfate ($Na_2S_2O_5$) (5 mL). The aqueous layer was further extracted with ethyl acetate (5 mL x 3), the combined organic extract was washed with brine (5 mL), dried over sodium sulphate, evaporated to obtain crude compound which was purified by silica gel column chromatography to afford epoxy ketone **6** as semi solid (43 mg, 82%). R_f= 0.6 (3:2 hexane/ethyl acetate); IR (neat):υ_{max} 2929, 2856, 1749, 1084, 839, 777 cm^{-1}; ^{1}H NMR (300 MHz, $CDCl_3$): δ 5.41 (m, 1H), 4.63 (q, *J* = 6.6 Hz, 2H), 4.30 (br s, 1H), 3.93 (d, *J* = 12.3 Hz, 1H), 3.81 (d, *J* = 11.9 Hz, 1H), 3.72 (d, *J* = 12.3 Hz, 1H), 3.56 (d, *J* = 11.9 Hz, 1H), 3.38 (s, 3H), 3.37 (s, 1H), 2.78 (d, *J* = 8.1 Hz, 1H), 2.53 (dd, *J* = 18.3, 8.1 Hz, 1H), 2.43 (d, *J* = 16.8 Hz, 1 H), 1.93 (d, *J* = 18.5 Hz, 1H), 1.77 (d, *J* = 17.4 Hz, 1H), 1.72 (s, 3H); ^{13}C NMR (75MHz, $CDCl_3$): δ 209.1, 143.1, 124.8, 96.7, 88.6, 72.4, 66.4, 66.3, 61.2, 55.5, 50.1, 40.8, 39.7, 35.1, 16.8; Mass (ESI): *m/z* [M+Na]$^+$ 303; HRMS (ESIMS) calcd for $C_{15}H_{20}O_5Na$ [M+Na]$^+$: *m/z* 303.1203; found: 303.1212.

<u>**^{1}H and ^{13}C NMR Spectra**</u>

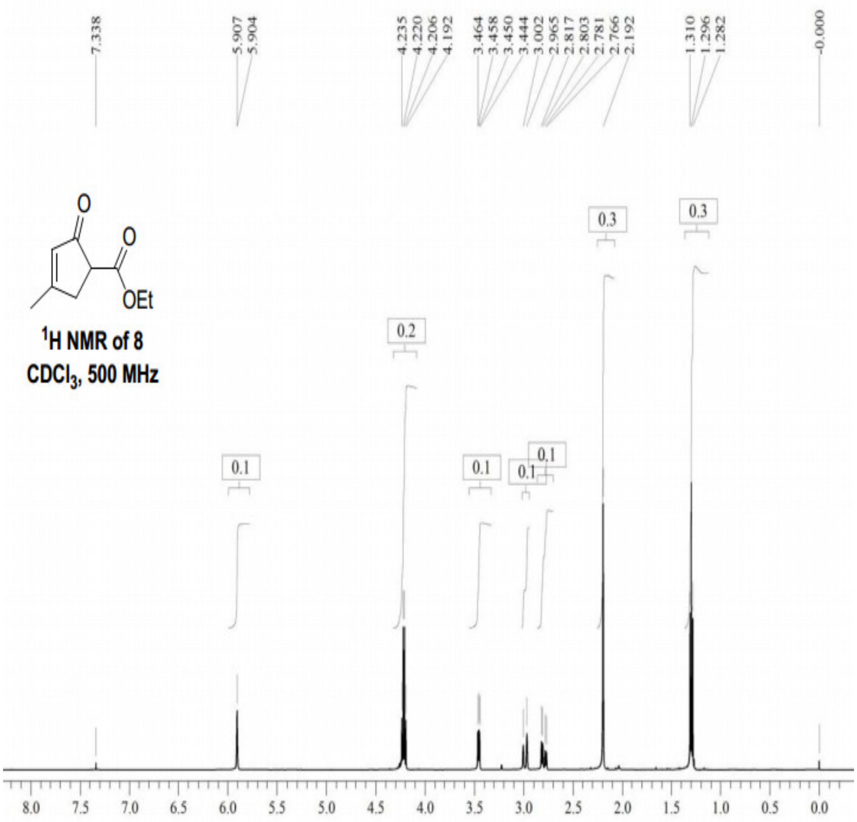

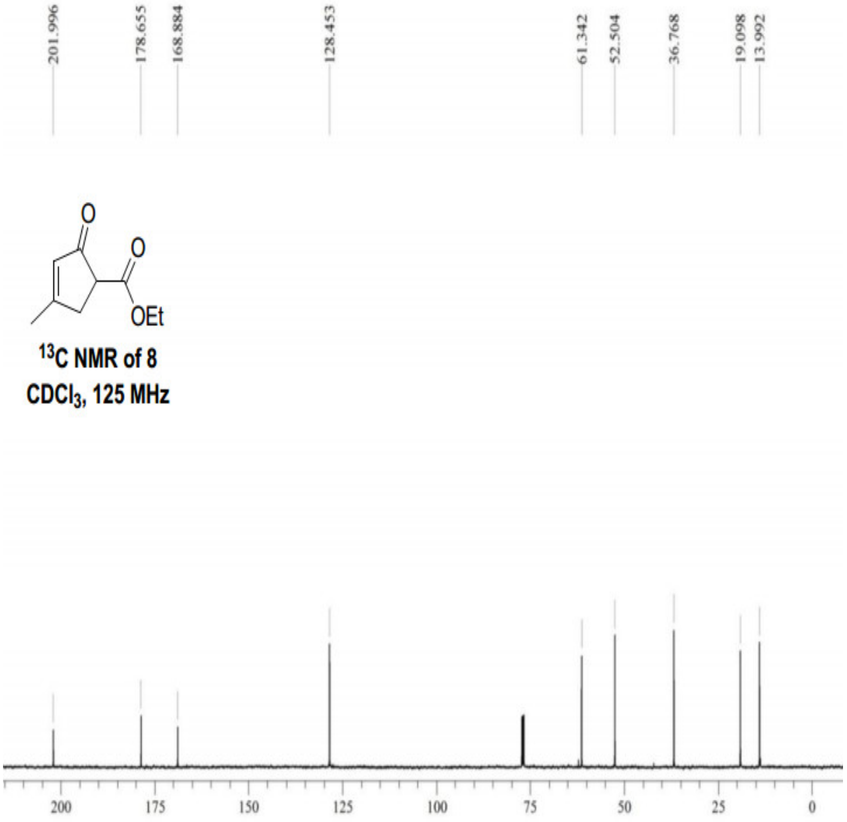

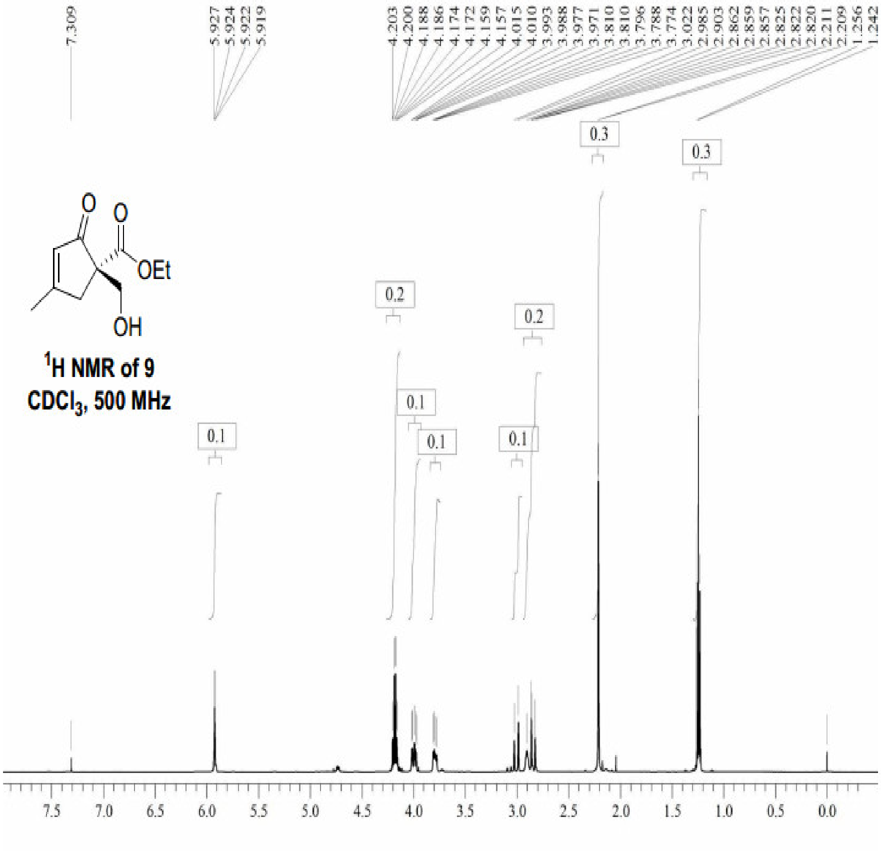
7.309
5.927 5.924 5.922 5.919
4.203 4.200 4.188 4.186 4.174 4.172 4.159 4.157 4.015 4.010 3.993 3.988 3.977 3.971 3.810 3.810 3.796 3.788 3.774 3.022 2.985 2.903 2.862 2.859 2.857 2.825 2.822 2.820 2.211 2.209 1.256 1.242
OEt
OH
^{1}H NMR of 9
$CDCl_3$, 500 MHz
0.1 0.2 0.1 0.1 0.1 0.2 0.3 0.3
7.5 7.0 6.5 6.0 5.5 5.0 4.5 4.0 3.5 3.0 2.5 2.0 1.5 1.0 0.5 0.0

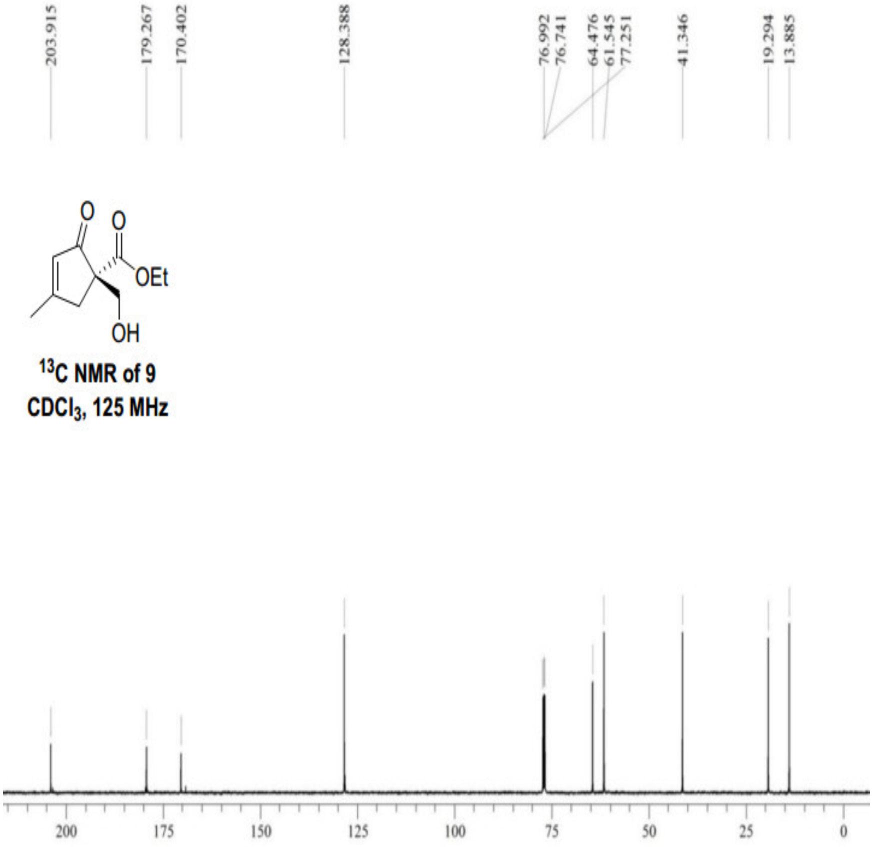
203.915
179.267
170.402
128.388
76.992 76.741 64.476 61.545 77.251
41.346
19.294 13.885
OEt
OH
^{13}C NMR of 9
$CDCl_3$, 125 MHz
200 175 150 125 100 75 50 25 0

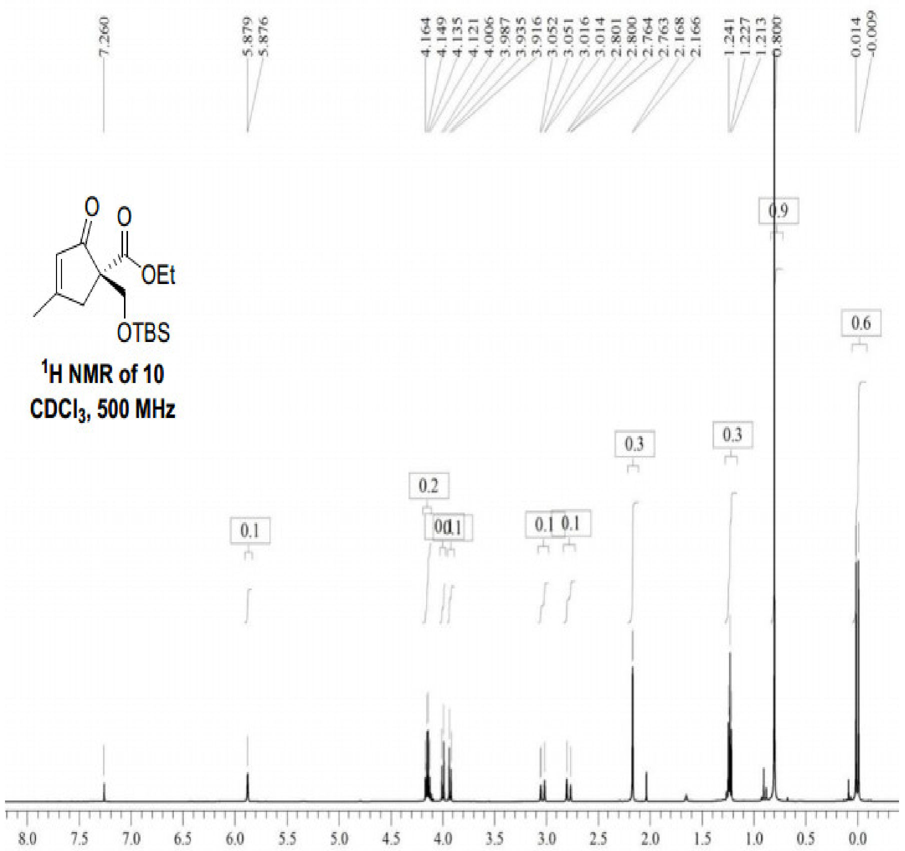

O
O
OEt
OTBS
1H NMR of 10
CDCl3, 500 MHz
7.260
5.879
5.876
4.164
4.149
4.135
4.121
4.006
3.987
3.935
3.916
3.052
3.051
3.016
3.014
2.801
2.800
2.764
2.763
2.168
2.166
1.241
1.227
1.213
0.800
0.014
-0.009
0.1
0.2
0.1
0.1
0.1
0.1
0.3
0.3
0.9
0.6
8.0
7.5
7.0
6.5
6.0
5.5
5.0
4.5
4.0
3.5
3.0
2.5
2.0
1.5
1.0
0.5
0.0

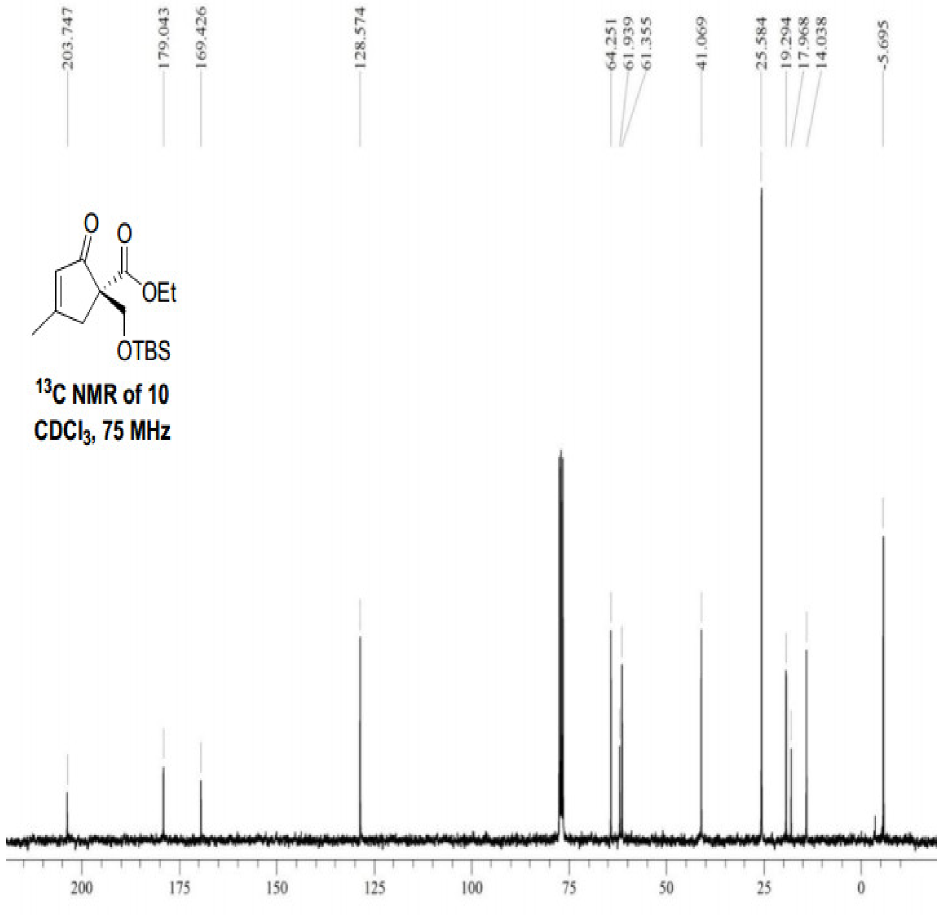

O
O
OEt
OTBS
13C NMR of 10
CDCl3, 75 MHz
203.747
179.043
169.426
128.574
64.251
61.939
61.355
41.069
25.584
19.294
17.968
14.038
-5.695
200
175
150
125
100
75
50
25
0

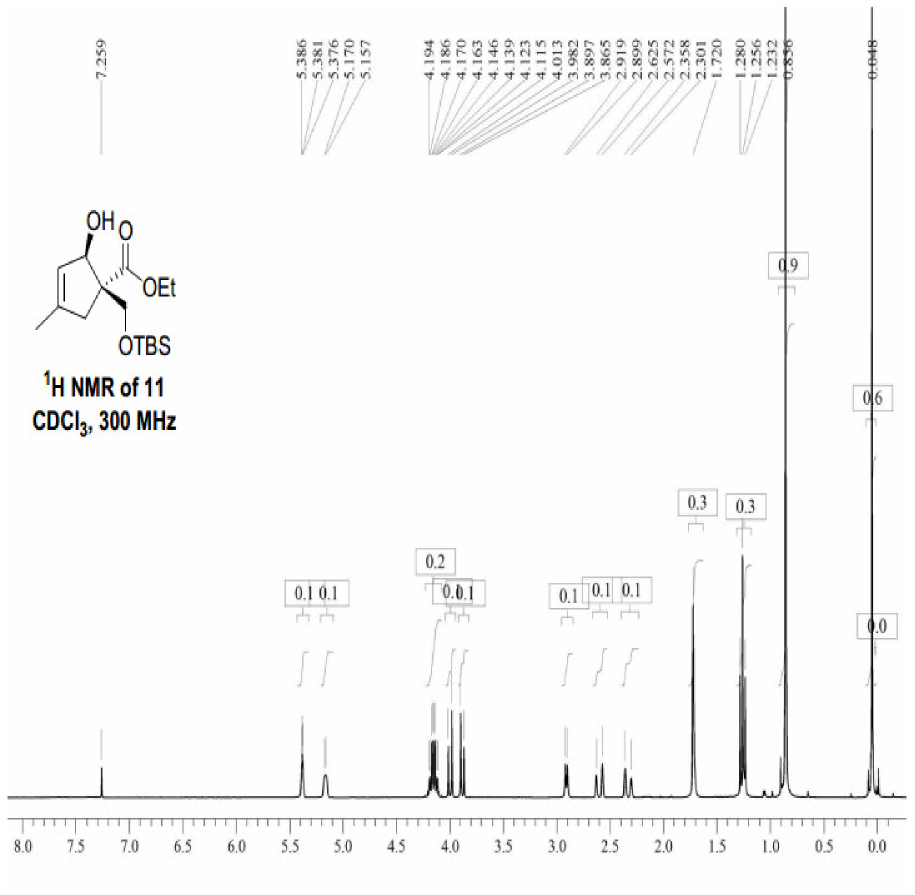
OH
O
OEt
OTBS
1H NMR of 11
CDCl3, 300 MHz
7.259
5.386
5.381
5.376
5.170
5.157
4.194
4.186
4.170
4.163
4.146
4.139
4.123
4.115
4.013
3.982
3.897
3.865
2.919
2.899
2.625
2.572
2.358
2.301
1.720
1.280
1.256
1.232
0.856
0.048
8.0 7.5 7.0 6.5 6.0 5.5 5.0 4.5 4.0 3.5 3.0 2.5 2.0 1.5 1.0 0.5 0.0

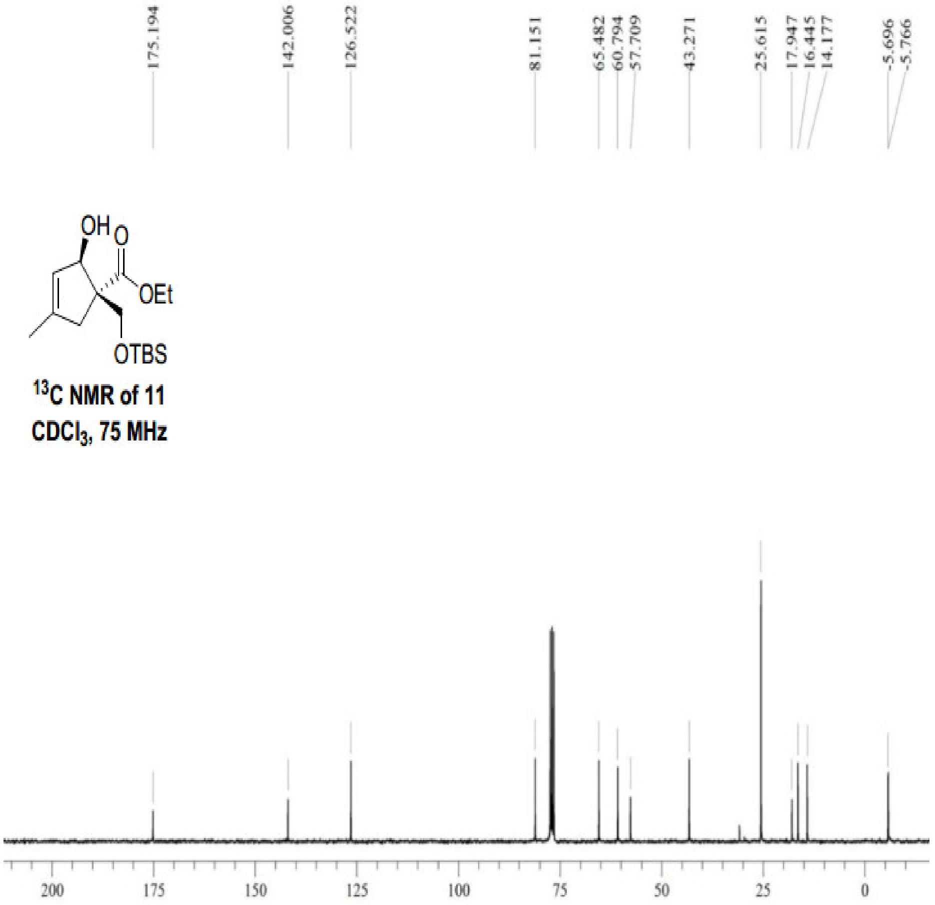
OH
O
OEt
OTBS
13C NMR of 11
CDCl3, 75 MHz
175.194
142.006
126.522
81.151
65.482
60.794
57.709
43.271
25.615
17.947
16.445
14.177
-5.696
-5.766
200 175 150 125 100 75 50 25 0

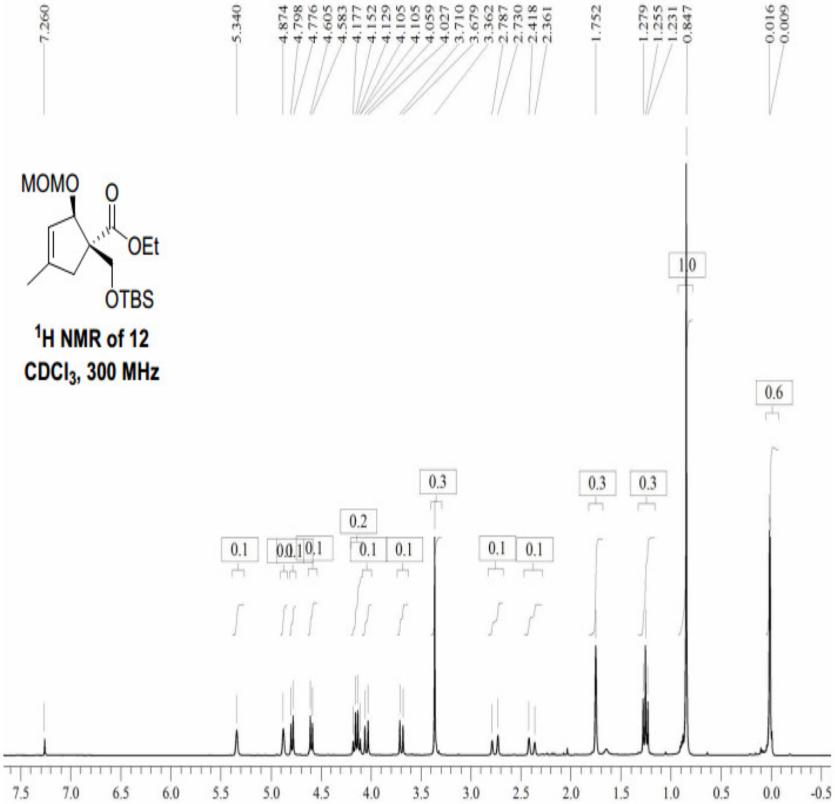
MOMO
O
OEt
OTBS
^{1}H NMR of 12
$CDCl_3$, 300 MHz
7.260 5.340 4.874 4.798 4.776 4.605 4.583 4.177 4.152 4.129 4.105 4.105 4.059 4.027 3.710 3.679 3.362 2.787 2.730 2.418 2.361 1.752 1.279 1.255 1.231 0.847 0.016 0.009
0.1 0.1 0.1 0.1 0.2 0.1 0.1 0.3 0.1 0.1 0.3 0.3 1.0 0.6
7.5 7.0 6.5 6.0 5.5 5.0 4.5 4.0 3.5 3.0 2.5 2.0 1.5 1.0 0.5 0.0 -0.5

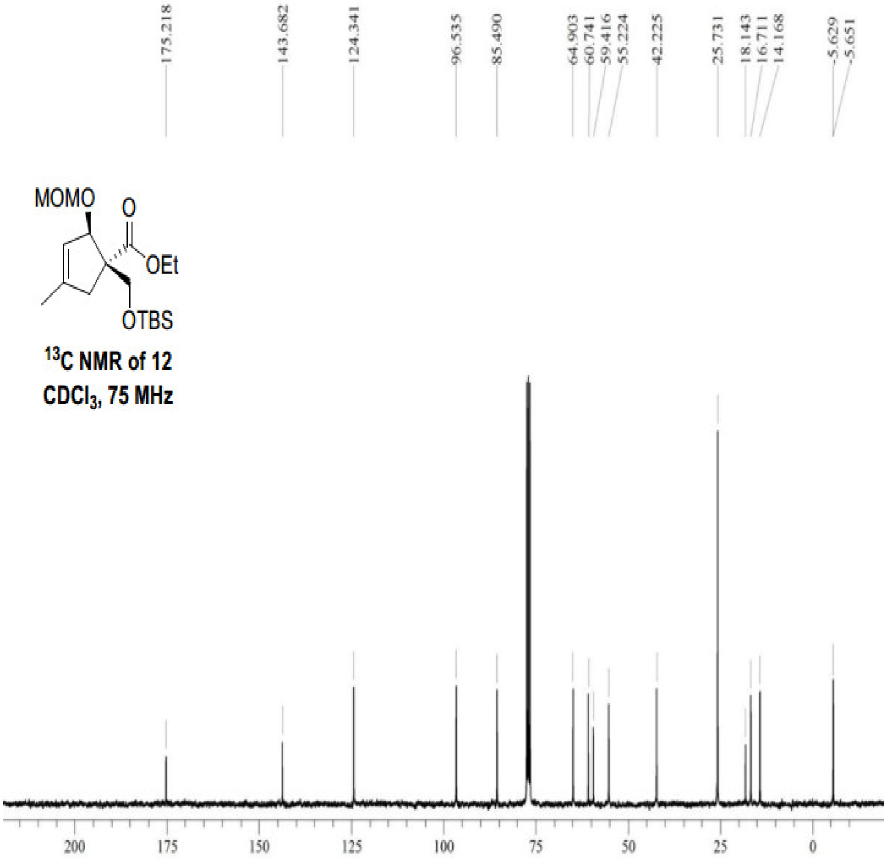
MOMO
O
OEt
OTBS
^{13}C NMR of 12
$CDCl_3$, 75 MHz
175.218 143.682 124.341 96.535 85.490 64.903 60.741 59.416 55.224 42.225 25.731 18.143 16.711 14.168 -5.629 -5.651
200 175 150 125 100 75 50 25 0

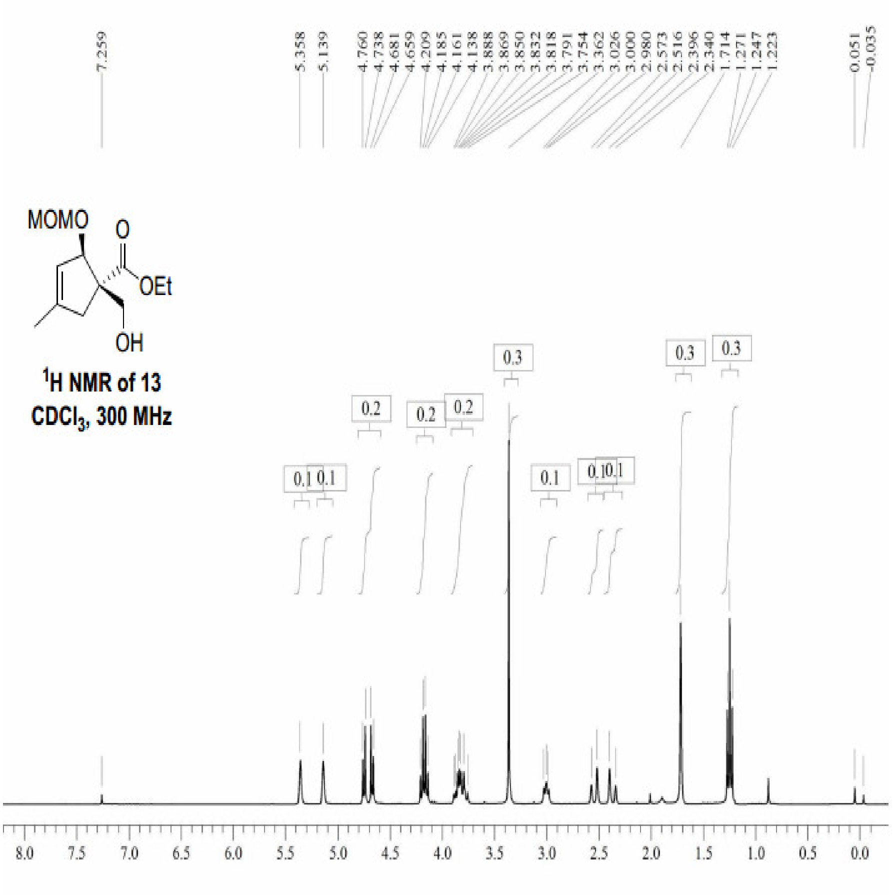
7.259
5.358
5.139
4.760
4.738
4.681
4.659
4.209
4.185
4.161
4.138
3.888
3.869
3.850
3.832
3.818
3.791
3.754
3.362
3.026
3.000
2.980
2.573
2.516
2.396
2.340
1.714
1.271
1.247
1.223
0.051
-0.035
MOMO
O
OEt
OH
^{1}H NMR of 13
$CDCl_3$, 300 MHz
0.1
0.1
0.2
0.2
0.2
0.3
0.1
0.1
0.1
0.3
0.3
8.0
7.5
7.0
6.5
6.0
5.5
5.0
4.5
4.0
3.5
3.0
2.5
2.0
1.5
1.0
0.5
0.0

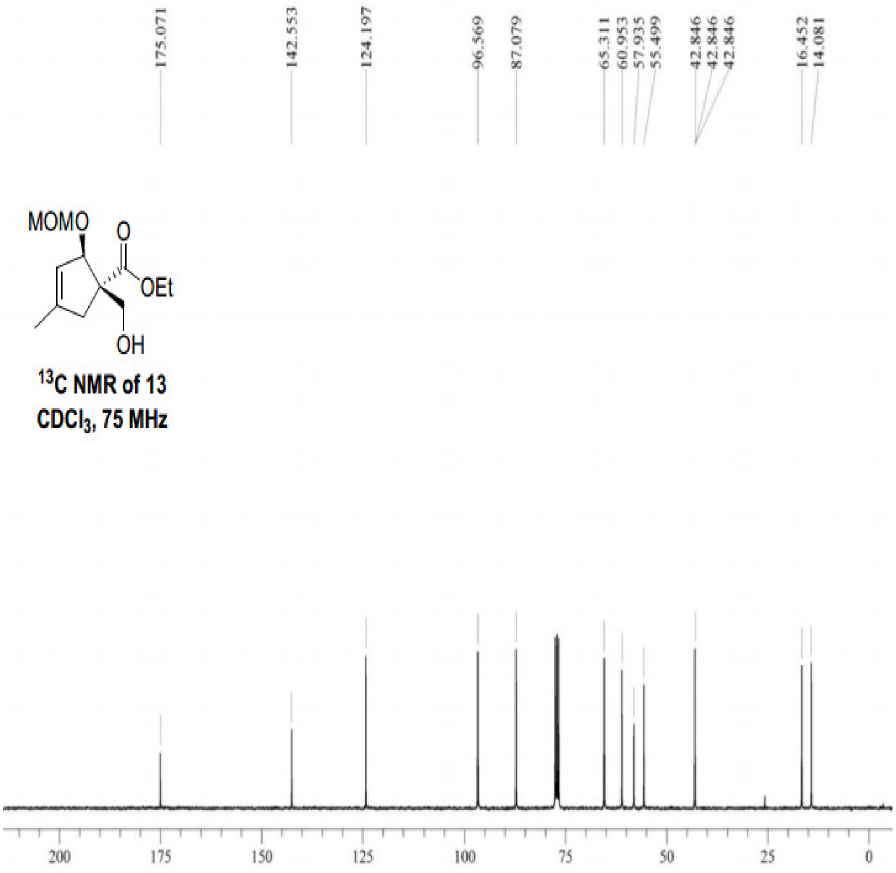
175.071
142.553
124.197
96.569
87.079
65.311
60.953
57.935
55.499
42.846
42.846
42.846
16.452
14.081
MOMO
O
OEt
OH
^{13}C NMR of 13
$CDCl_3$, 75 MHz
200
175
150
125
100
75
50
25
0

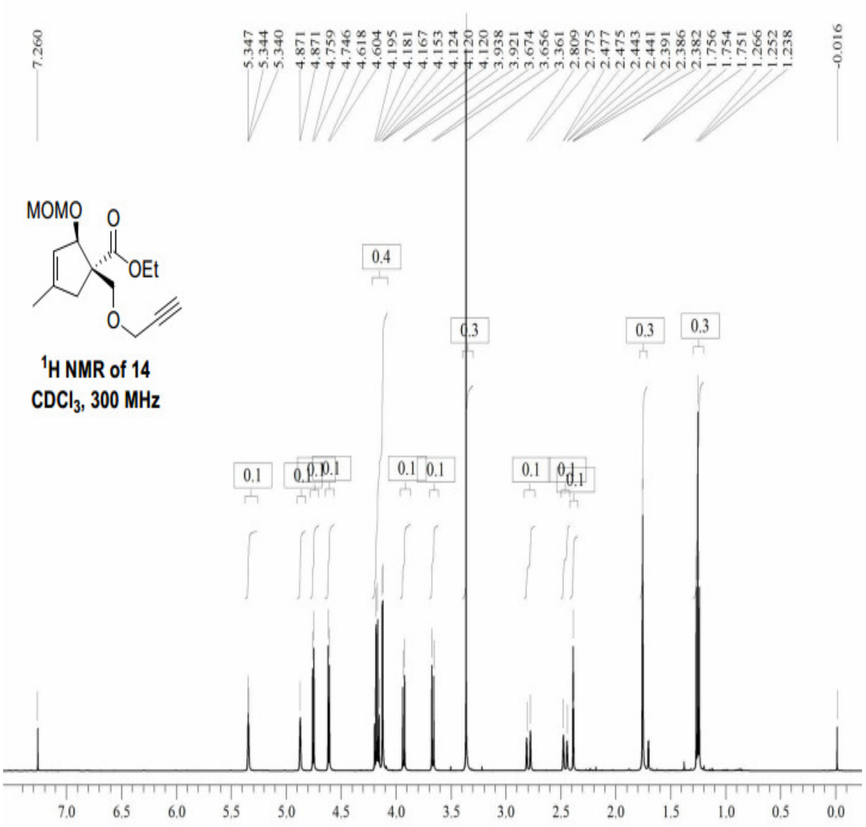

^{1}H NMR of 14
$CDCl_3$, 300 MHz

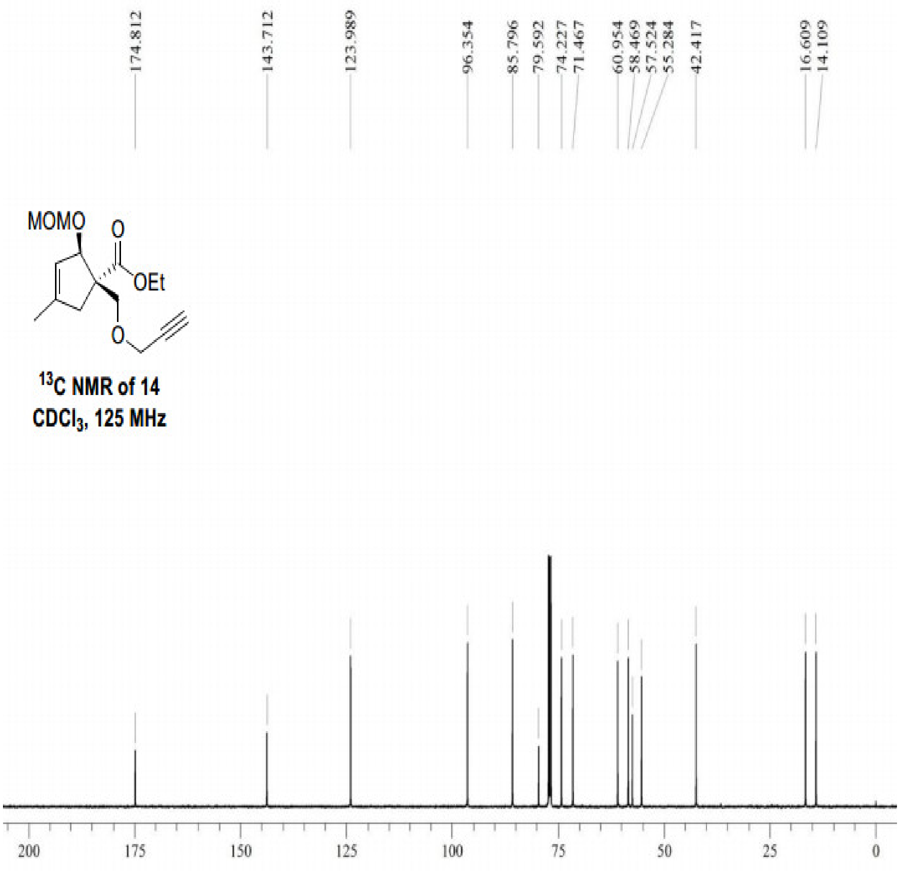

^{13}C NMR of 14
$CDCl_3$, 125 MHz

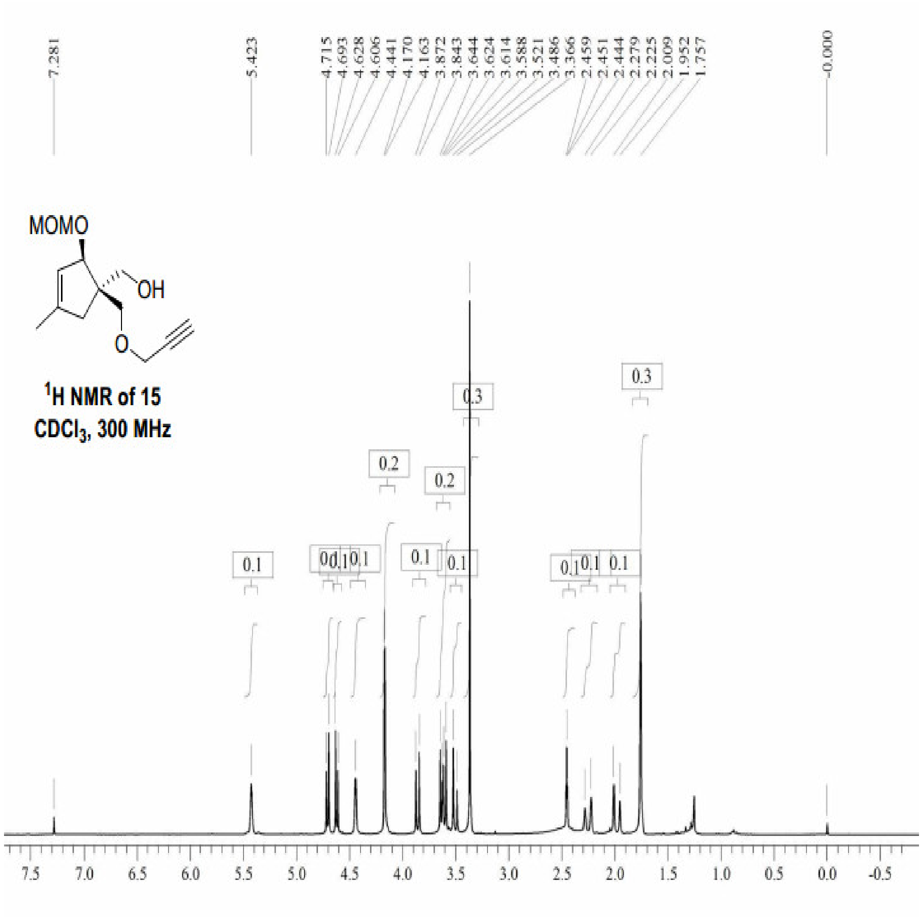
MOMO
OH
O
1H NMR of 15
CDCl3, 300 MHz
7.281
5.423
4.715
4.693
4.628
4.606
4.441
4.170
4.163
3.872
3.843
3.644
3.624
3.614
3.588
3.521
3.486
3.366
2.459
2.451
2.444
2.279
2.225
2.009
1.952
1.757
-0.000

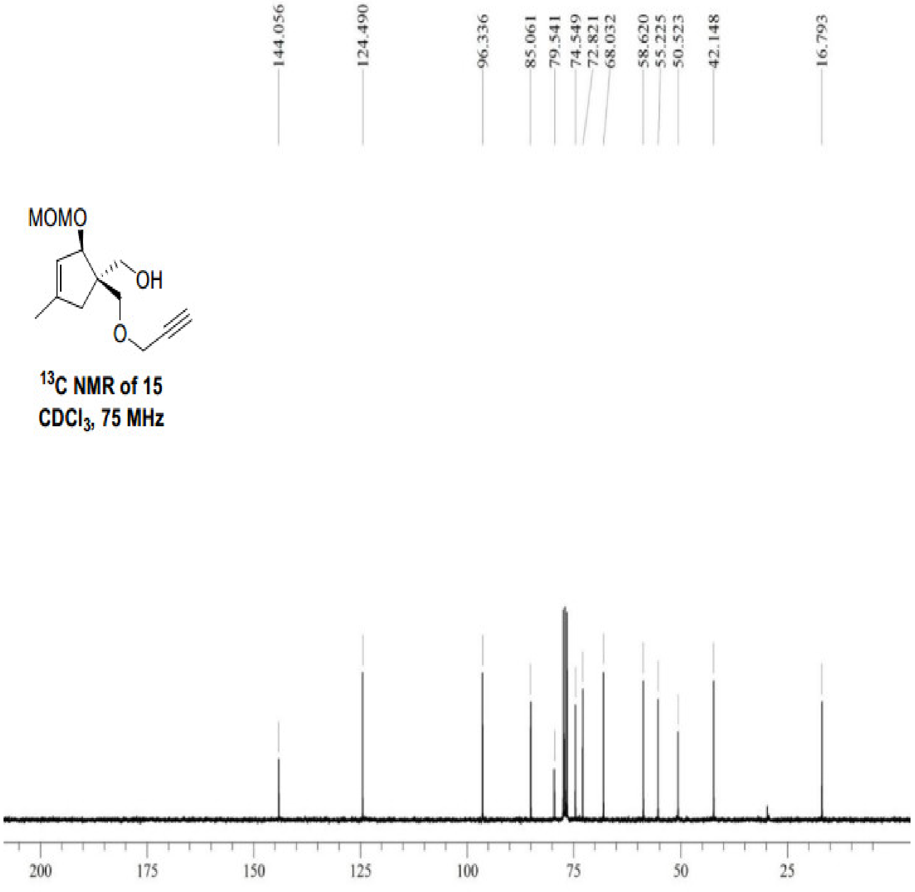
MOMO
OH
O
13C NMR of 15
CDCl3, 75 MHz
144.056
124.490
96.336
85.061
79.541
74.549
72.821
68.032
58.620
55.225
50.523
42.148
16.793

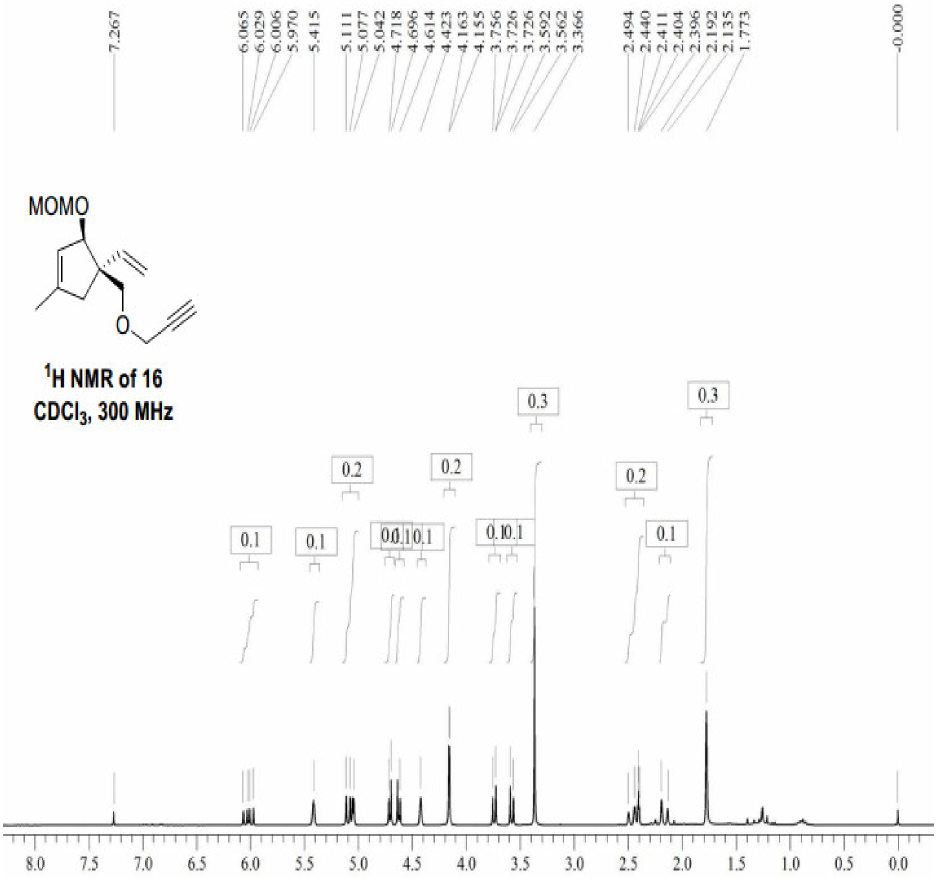

7.267
6.065
6.029
6.006
5.970
5.415
5.111
5.077
5.042
4.718
4.696
4.614
4.423
4.163
4.155
3.756
3.726
3.726
3.592
3.562
3.366
2.494
2.440
2.411
2.404
2.396
2.192
2.135
1.773
-0.000
MOMO
O
^{1}H NMR of 16
CDCl$_3$, 300 MHz
8.0
7.5
7.0
6.5
6.0
5.5
5.0
4.5
4.0
3.5
3.0
2.5
2.0
1.5
1.0
0.5
0.0

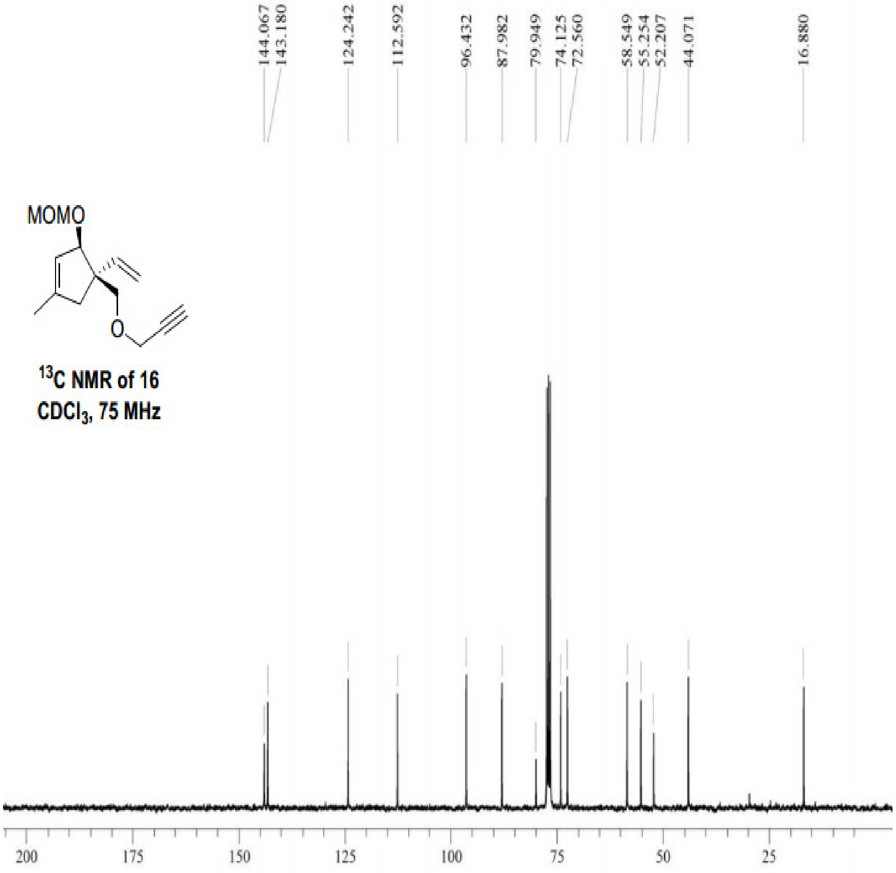

144.067
143.180
124.242
112.592
96.432
87.982
79.949
74.125
72.560
58.549
55.254
52.207
44.071
16.880
MOMO
O
^{13}C NMR of 16
CDCl$_3$, 75 MHz
200
175
150
125
100
75
50
25

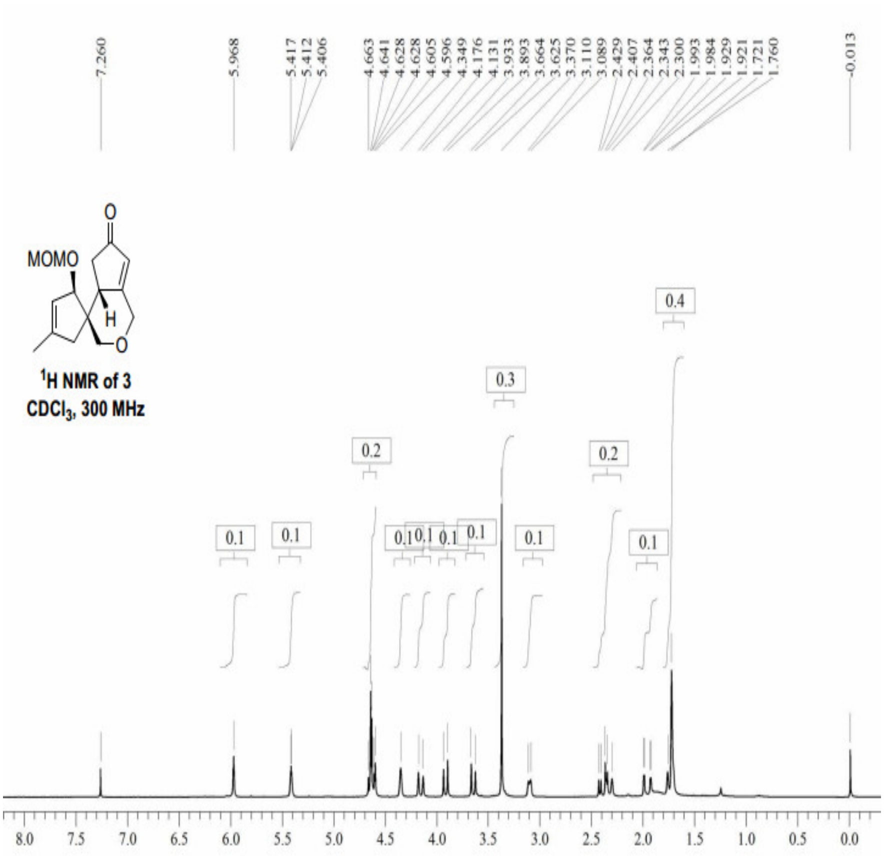
7.260
5.968
5.417
5.412
5.406
4.663
4.641
4.628
4.628
4.605
4.596
4.349
4.176
4.131
3.933
3.893
3.664
3.625
3.370
3.110
3.089
2.429
2.407
2.364
2.343
2.300
1.993
1.984
1.929
1.921
1.721
1.760
-0.013
MOMO
H
O
^{1}H NMR of 3
$CDCl_3$, 300 MHz
0.1
0.1
0.2
0.1
0.1
0.1
0.1
0.3
0.1
0.2
0.1
0.4
8.0 7.5 7.0 6.5 6.0 5.5 5.0 4.5 4.0 3.5 3.0 2.5 2.0 1.5 1.0 0.5 0.0

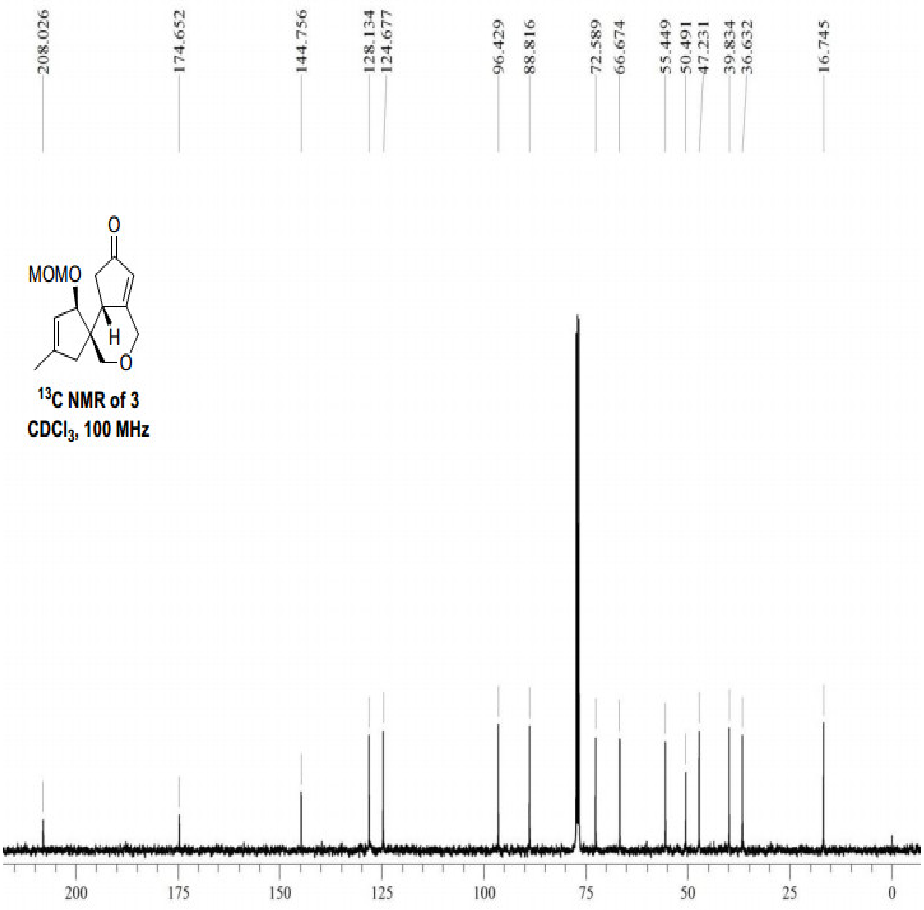
208.026
174.652
144.756
128.134
124.677
96.429
88.816
72.589
66.674
55.449
50.491
47.231
39.834
36.632
16.745
MOMO
H
O
^{13}C NMR of 3
$CDCl_3$, 100 MHz
200 175 150 125 100 75 50 25 0

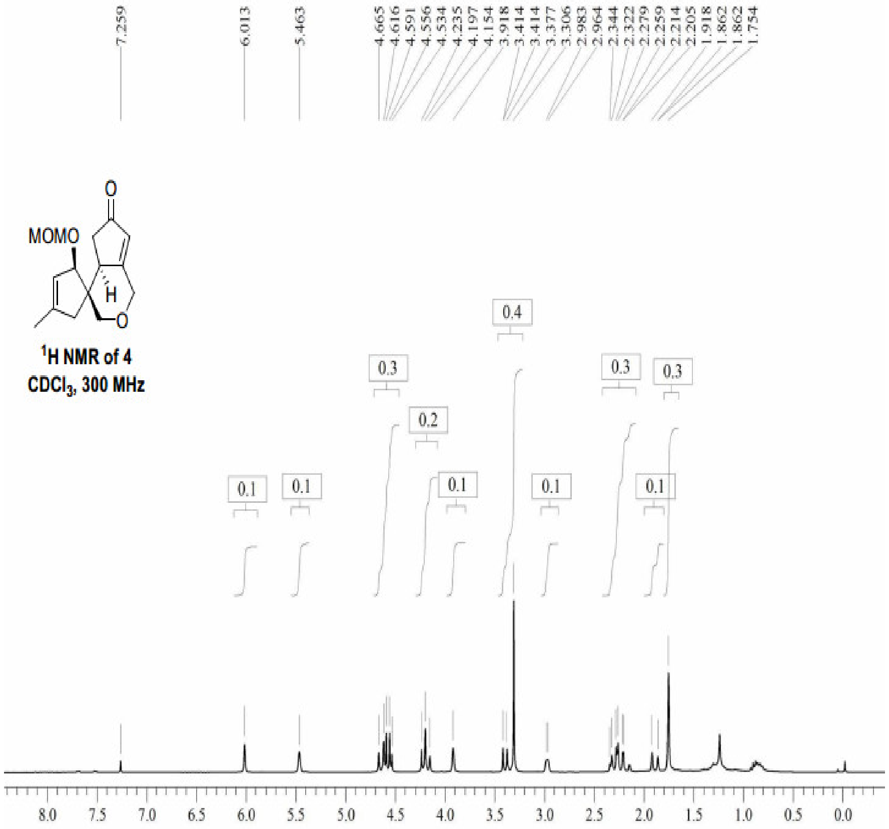

MOMO
O
H
^{1}H NMR of 4
$CDCl_3$, 300 MHz
7.259
6.013
5.463
4.665
4.616
4.591
4.556
4.534
4.235
4.197
4.154
3.918
3.414
3.414
3.377
3.306
2.983
2.964
2.344
2.322
2.279
2.259
2.214
2.205
1.918
1.862
1.862
1.754
0.1
0.1
0.3
0.2
0.1
0.4
0.1
0.3
0.1
0.3
8.0
7.5
7.0
6.5
6.0
5.5
5.0
4.5
4.0
3.5
3.0
2.5
2.0
1.5
1.0
0.5
0.0

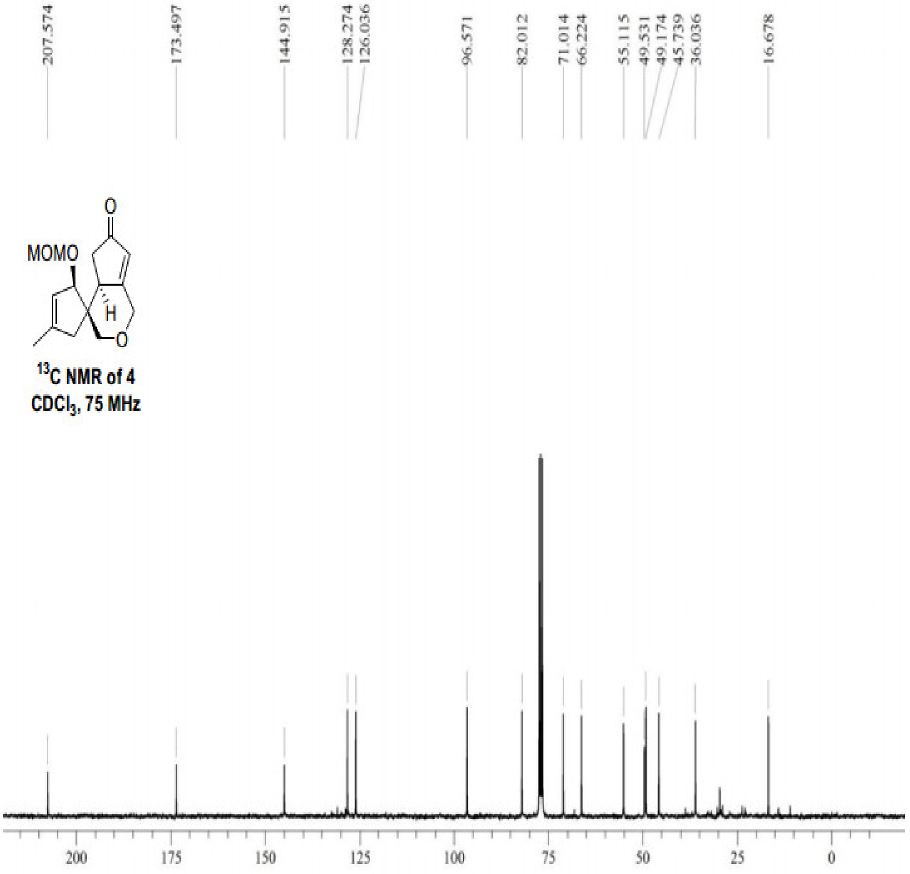

MOMO
O
H
^{13}C NMR of 4
$CDCl_3$, 75 MHz
207.574
173.497
144.915
128.274
126.036
96.571
82.012
71.014
66.224
55.115
49.531
49.174
45.739
36.036
16.678
200
175
150
125
100
75
50
25
0

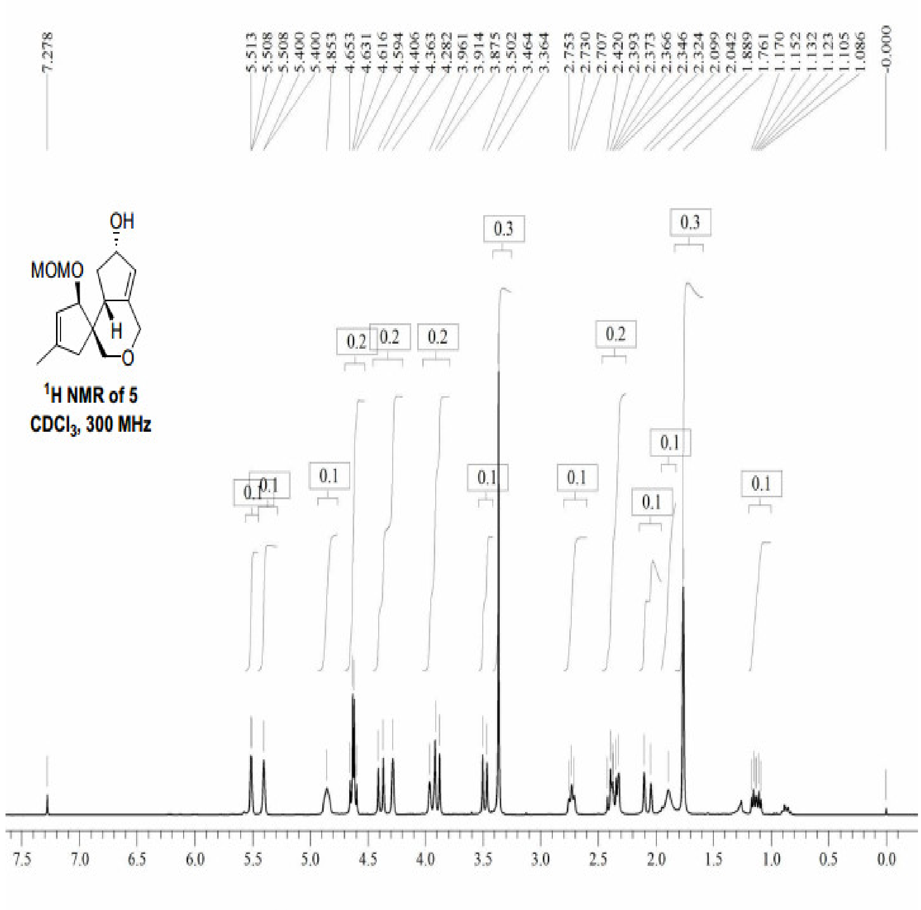

7.278
5.513 5.508 5.508 5.400 5.400 4.853 4.653 4.631 4.616 4.594 4.406 4.363 4.282 3.961 3.914 3.875 3.502 3.464 3.364
2.753 2.730 2.707 2.420 2.393 2.373 2.366 2.346 2.324 2.099 2.042 1.889 1.761 1.170 1.152 1.132 1.123 1.105 1.086
-0.000
OH
MOMO
H
O
^{1}H NMR of 5
$CDCl_3$, 300 MHz
0.1 0.1 0.1 0.2 0.2 0.2 0.1 0.3 0.1 0.2 0.1 0.1 0.3 0.1
7.5 7.0 6.5 6.0 5.5 5.0 4.5 4.0 3.5 3.0 2.5 2.0 1.5 1.0 0.5 0.0

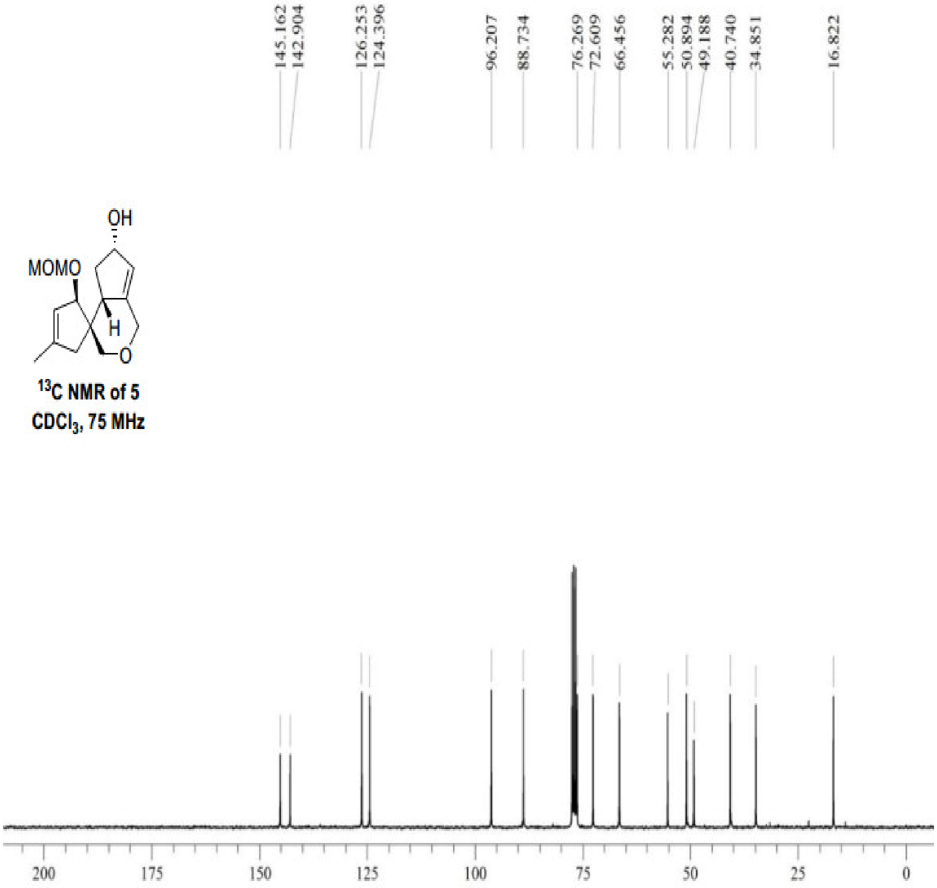

145.162 142.904 126.253 124.396 96.207 88.734 76.269 72.609 66.456 55.282 50.894 49.188 40.740 34.851 16.822
OH
MOMO
H
O
^{13}C NMR of 5
$CDCl_3$, 75 MHz
200 175 150 125 100 75 50 25 0

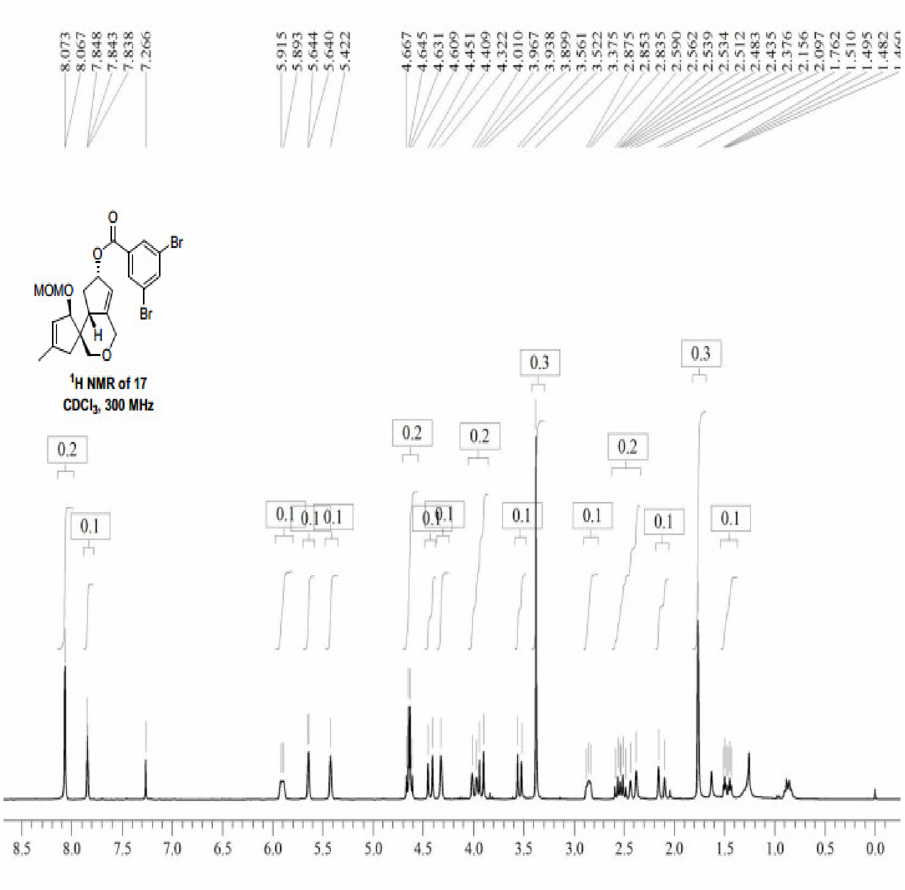
8.073
8.067
7.848
7.843
7.838
7.266
5.915
5.893
5.644
5.640
5.422
4.667
4.645
4.631
4.609
4.451
4.409
4.322
4.010
3.967
3.938
3.899
3.561
3.522
3.375
2.875
2.853
2.835
2.590
2.562
2.539
2.534
2.512
2.483
2.435
2.376
2.156
2.097
1.762
1.510
1.495
1.482
MOMO
O
Br
Br
H
O
¹H NMR of 17
CDCl₃, 300 MHz
0.2
0.1
0.3
8.5 8.0 7.5 7.0 6.5 6.0 5.5 5.0 4.5 4.0 3.5 3.0 2.5 2.0 1.5 1.0 0.5 0.0
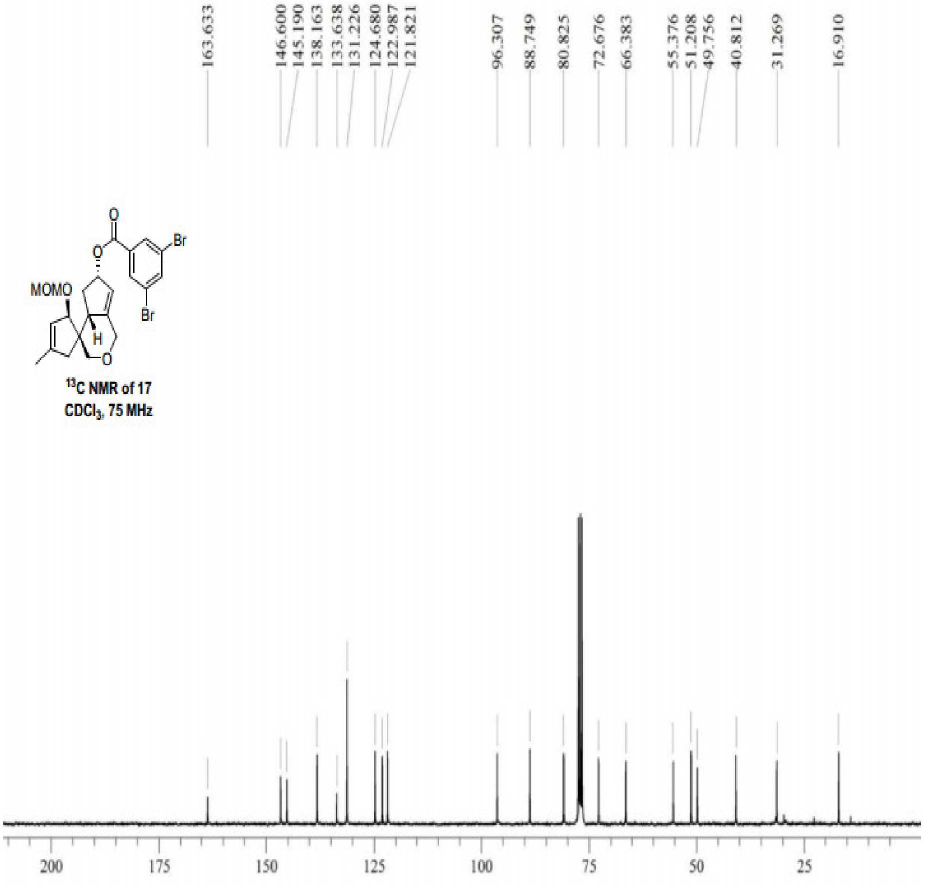
163.633
146.600
145.190
138.163
133.638
131.226
124.680
122.987
121.821
96.307
88.749
80.825
72.676
66.383
55.376
51.208
49.756
40.812
31.269
16.910
MOMO
O
Br
Br
H
O
¹³C NMR of 17
CDCl₃, 75 MHz
200 175 150 125 100 75 50 25

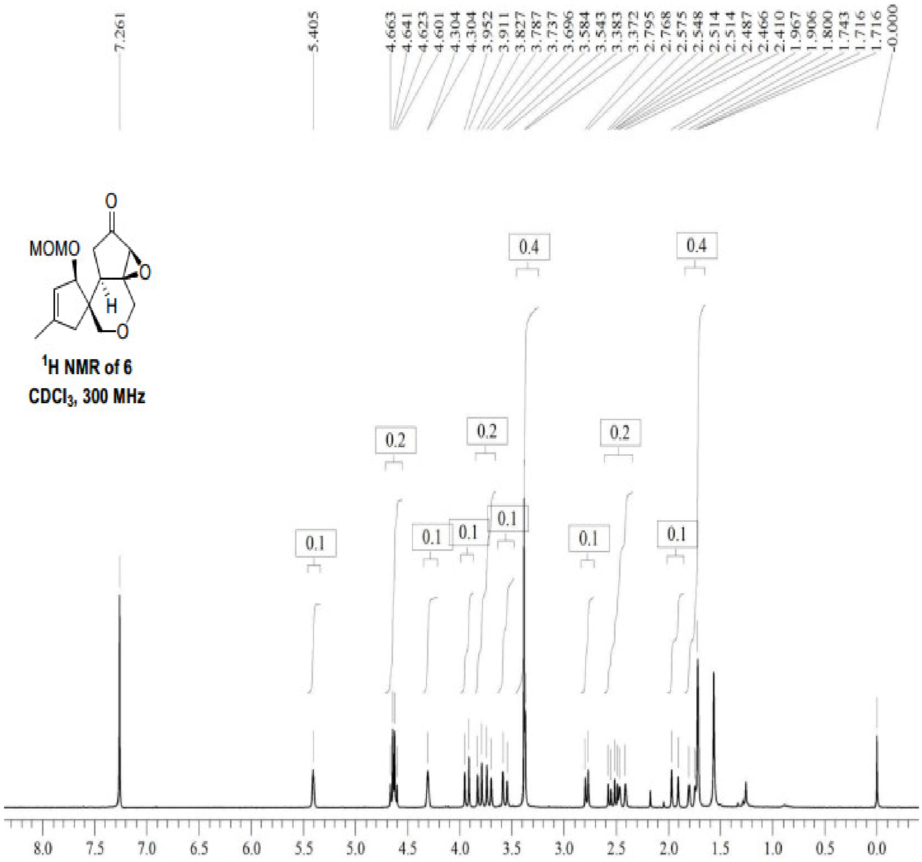
MOMO
O
H
^{1}H NMR of 6
$CDCl_3$, 300 MHz
7.261
5.405
4.663 4.641 4.623 4.601 4.304 4.304 3.952 3.911 3.827 3.787 3.737 3.696 3.584 3.543 3.383 3.372 2.795 2.768 2.575 2.548 2.514 2.514 2.487 2.466 2.410 1.967 1.906 1.800 1.743 1.716 1.716 -0.000

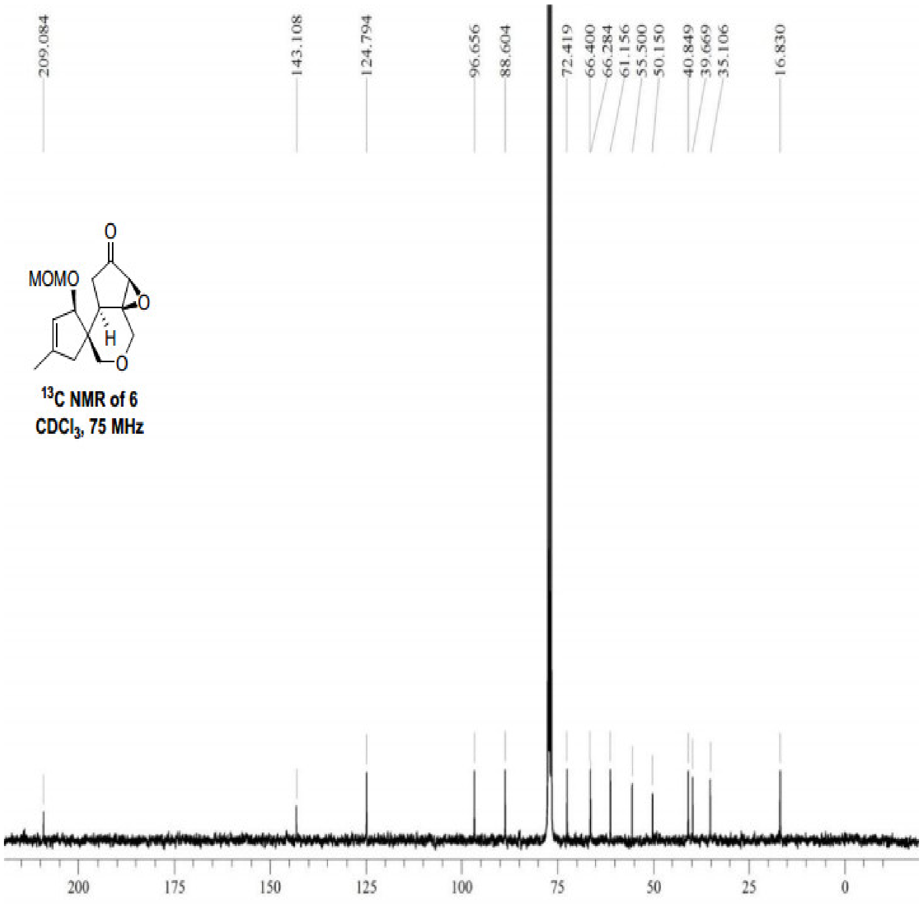
MOMO
O
H
^{13}C NMR of 6
$CDCl_3$, 75 MHz
209.084
143.108
124.794
96.656
88.604
72.419 66.400 66.284 61.156 55.500 50.150 40.849 39.669 35.106
16.830
